# *C*- and *N*-Phosphorylated Enamines—An Avenue to Heterocycles: NMR Spectroscopy

**DOI:** 10.3390/ijms24119646

**Published:** 2023-06-01

**Authors:** Lyudmila Larina

**Affiliations:** A. E. Favorsky Irkutsk Institute of Chemistry, Siberian Branch of the Russian Academy of Sciences, 1 Favorsky St., 664033 Irkutsk, Russia; larina@irioch.irk.ru

**Keywords:** *C*- and *N*-chlorophosphorylated enamines, nitrogen–phosphorus-containing heterocycles, phosphorus pentachloride, *E*- and *Z*-isomers, structure, 2D and ^1^H, ^13^C and ^31^P NMR spectroscopy, ^35^Cl NQR spectroscopy

## Abstract

The review presents extensive data (from the works of the author and literature) on the structure of *C*- and *N*-chlorophosphorylated enamines and the related heterocycles obtained by multipulse multinuclear ^1^H, ^13^C, and ^31^P NMR spectroscopy. The use of phosphorus pentachloride as a phosphorylating agent for functional enamines enables the synthesis of various *C*- and *N*-phosphorylated products that are heterocyclized to form various promising nitrogen- and phosphorus-containing heterocyclic systems. ^31^P NMR spectroscopy is the most convenient, reliable and unambiguous method for the study and identification of organophosphorus compounds with different coordination numbers of the phosphorus atom, as well as for the determination of their *Z*- and *E*-isomeric forms. An alteration of the coordination number of the phosphorus atom in the phosphorylated compounds from 3 to 6 leads to a drastic screening of the ^31^P nucleus from about +200 to −300 ppm. The unique structural features of nitrogen–phosphorus-containing heterocyclic compounds are discussed.

## 1. Introduction

The chemistry of organophosphorus compounds (OPCs) is one of the rapidly developing areas of organic chemistry. Phosphorus compounds have attracted much interest in the past decades due to their wide applications ranging from synthetic chemistry to materials science and life sciences. Interest in organophosphorus compounds is due to their importance for the chemistry of macromolecular materials, complexones, chelating agents, extragents, antipyrenes, drugs, phosphorus-containing pesticides and other materials [1,2,3,4,5,6,7,8,9]. Due to a wide range of physiological activity and easy degradation into the simplest non-toxic products, organophosphorus compounds rank among the best plant protection chemicals. Although organophosphorus chemistry is known as a mature and specialized field, researchers would like to develop new methods for the synthesis of OPCs to improve the safety and sustainability of chemical processes [10,11,12,13].

A well-known method for obtaining unsaturated OPCs having phosphorus chloride groups at the double bond is the reaction of phosphorus pentachloride with various organic nucleophiles—alkenes, alkadienes, alkynes, ethers and esters, tertiary amines and other compounds [14,15,16,17,18,19,20]. The availability of the starting reagents, mild reaction conditions and the ability to vary the structure of the final OPCs are advantages of this method. This reaction has not exhausted its synthetic capabilities in terms of the covering of new nucleophiles. Therefore, research in this area still remains a challenge.

Organic derivatives of phosphorus pentachloride in various coordination states, including the products of phosphorylation of alkenes and heterocycles based on them, can be conveniently studied by ^31^P NMR spectroscopy. As is known, phosphorus pentachloride, a common electrophilic phosphorylating agent, dissociates into ions both in the crystalline state and in polar solvents and exists in a pentacoordinated state in nonpolar solvents:

$$2\;PCl_{5}⇌PCl_{4}^{+}+PCl_{6}^{-}$$

A variation in the coordination number of phosphorus from 3 to 6 significantly changes the shielding of the ^31^P nucleus from about +200 to −300 ppm:
Phosphorous chloride${\mathrm{PCl}}_{3}$${\mathrm{PCI}}_{4}^{+}$${\mathrm{PCl}}_{5}$${\mathrm{PCl}}_{6}^{-}$Coordination number3456^31^P NMR chemical shift, pm+200+80−80−300

The tetra-, penta- and hexacoordinated states of phosphorus pentachloride can be very easily characterized by multinuclear multipulse NMR spectroscopy and, in particular, by ^31^P NMR spectroscopy [9]. 

The phosphorylation reaction of a number of nitrogen-containing organic nucleophiles involves the electrophilic addition of phosphorus pentachloride to the double bond of enamines introduced into the reaction or formed during the phosphorylation of enamides, amides, ureides, tertiary amines and other nitrogen-containing organic nucleophiles. Phosphorus-containing enamines and enamides successfully combine a double bond and phosphorus and nitrogen organic fragments in a molecule. Due to the presence of several reaction centers, they are key compounds in the synthesis of promising functionalized heterocycles. In addition, organic compounds containing phosphorus and nitrogen at the same time are interesting objects for studying conjugation, stereochemistry and geometric isomerism. 

The stereochemistry of functionalized enamines incorporating phosphorus atoms is a challenging topic in heteroatom chemistry because the correct interpretation of their chemical behavior and biological activity depends on understanding the factors that determine the stereochemical features and relative stability of their isomers. 

The structural aspects of phosphorylated unsaturated compounds, such as *N*-vinylazoles and their derivatives, are considered in detail in the review [9]. It has been shown that many *N*-vinylazoles react with phosphorus pentachloride to form organophosphorus compounds containing tetra-, penta- and hexacoordinated phosphorus atoms.

## 2. The Phosphorylated Tertiary Amines

Tertiary amines are known to react intensely with phosphorus chlorides. This often leads to the resinification of the reaction mixture and does not allow the reaction products to be isolated as individual compounds. The reactions of tertiary amines with phosphorus chlorides deliver donor–acceptor complexes followed by the chlorination of alkyl groups, which in most cases leads to iminium salts with the N–C bond cleavage [21,22,23]. In the presence of excess tertiary amine, iminium salts are transformed into enamines, which can undergo *C*-phosphorylation with phosphorus pentachloride.

**Scheme 1:**
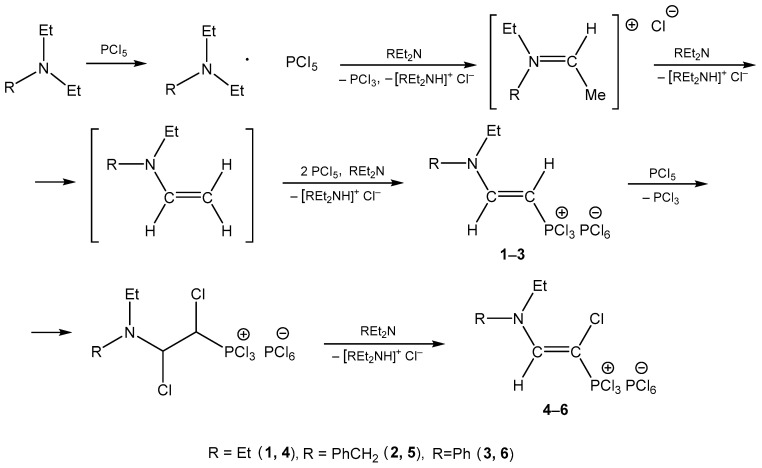
The formation of hexachlorophosphorates of 2-aminoethenyltrichloro-phosphonium (**1**–**3**) and hexachlorophosphorates of 2-amino-1-chloroethenyltrichlorophosphorates (**4**–**6**).

The phosphorylation of tertiary *N*-ethylamines with phosphorus pentachloride has been studied in detail by two-dimensional (2D) and multinuclear ^1^H, ^13^C and ^31^P NMR spectroscopy [14,24,25]. The tertiary amines, containing at least two ethyl groups (triethylamine, diethylaniline, diethylbenzylamine), react with phosphorus pentachloride under mild conditions (15–20 °C) to form hexachlorophosphorates of 2-aminoethenyltrichlorophosphonium (**1**–**3**) and hexachlorophosphorates of 2-amino-1-chloroethenyltrichlorophosphorates (**4**–**6**). The following sequence of transformations was suggested to explain the formation of compounds **1**–**6** (Scheme 1).

The reaction is initiated by the formation of donor–acceptor complexes of tertiary amines with phosphorus pentachlorides, followed by chlorination of the complex compounds at the methylene group in the α-position to the nitrogen atom. Due to the instability of tertiary chloramines, they lose chlorine in the form of an anion, which leads to the formation of iminium salts. In the presence of excess tertiary amine, iminium salts are dehydrochlorinated into enamines, the latter being phosphorylated with phosphorus pentachloride. Phosphorylated enamines (**1**–**3**), obtained from tertiary amines due to the high basicity of dialkyl- and alkyl(aryl)amino groups, have an increased nucleophilicity of the double bond and are easily chlorinated by phosphorus pentachloride at the double bond, in contrast to phosphorylated derivatives of *N*-vinylheterocycles, for example, *N*-vinylbenzotriazole and *N*-vinylcarbazole [9,26,27]. The chlorination products are easily dehydrochlorinated due to the high C-H acidity of protons (P–CH=) with the formation of hexachlorophosphorates **4**–**6** (Scheme 1, Table 1). 

This was confirmed by studies of the reaction mixture by ^31^P NMR spectroscopy [24]. For example, two signals are observed in ^31^P NMR spectrum of the reaction products of phosphorus pentachloride with triethylamine: a doublet of doublets at 89.9 ppm ^2^*J*_PH_ 35.0 Hz and ^3^*J*_PH_ 25.9 Hz and a doublet at 80.7 ppm ^3^*J*_PH_ 14.1 Hz assigned to organyltrichlorophosphonium cations **1** and **4** (Table 1).

The amount of hexachlorophosphorate **1** in the reaction mixture in the ^31^P NMR data decreases in time, while that of chlorenamine **4** grows. This testifies that enamine **4** is formed from hexachlorophosphate **1**. The phosphorylation process is completed after the formation of enamine (**4**).

**Scheme 2:**
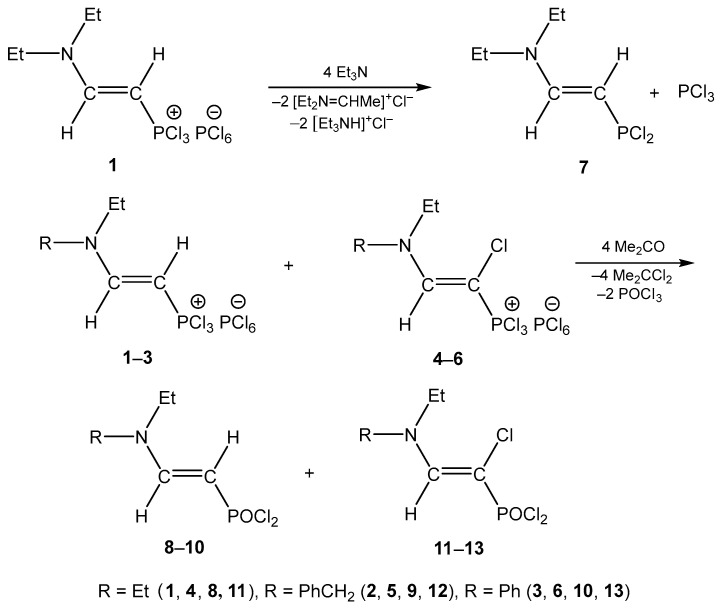
The formation of 2-(diethylamino)ethenylphosphonius acid (**7**) and transformation of enaminotrichlorophosphonium hexachlorophosphates (**1**–**6**) into dichloroanhydrides of phosphonous acids (**8**–**13**).

Phosphorylation of triethylamine in the presence of excess amine led to the arising in the reaction mixture of dichloroanhydride of 2-(diethylamino)-ethenylphosphonius acid (**7**), which was indicated by the presence of the signal at 160 ppm in the ^31^P NMR spectrum. Apparently, compound **7** is obtained through the reduction of 2-aminoethenyl-1-trichlorophosphonium hexachlorophosphate (**1**) with excess amine (Scheme 2).

It is not possible to separate crystalline hexachlorophosphates (**1**–**6**) from triorganylammonium chlorides presented in the reaction mixture, since the solubility of compounds **1**–**6** in organic solvents is close to that of triorganylammonium salts. This poses a serious preparative problem in the studies of the phosphorylation of tertiary amines with phosphorus pentachloride. Phosphorylation products of tertiary amines can be isolated in pure form by converting enaminotrichlorophosphonium hexachlorophosphates into phosphonic dichlorides **8**–**13**, which is achieved by treating hexachlorophosphates with sulfur dioxide or dry acetone (Scheme 2). As hydrochlorides of tertiary amines are insoluble in diethyl ether, and chloroanhydrides of phosphonous acids are soluble, compounds **8**–**13** have been isolated in pure form by extraction with diethyl ether. The structure of phosphorus-containing enamines (**1**–**6**, **8**–**13**) has been characterized by ^1^H, ^13^C and ^31^P NMR spectroscopy (Table 1).

The values of coupling constants ^3^*J*_HH_ 13–14 Hz for the phosphorylated enamines having both vinyl protons indicate the *trans*-orientation of protons in the molecule. The ^31^P NMR spectra of phosphorylated chlorenamines are characterized by the coupling constant ^3^*J*_PH_ values being decreased by 7–10 Hz as compared to phosphorylated enamines, containing no chlorine atom at the double bond. This fact is widely used to identify chlorophosphorylated compounds in organic synthesis. 

The presence of a chlorine atom exactly at the carbon atom, nearest to the phosphorus-containing substituent in compounds **4**–**6** and **11**–**13**, is unambiguously confirmed by ^13^C NMR spectral data (Table 1). The high value of coupling constants ^1^*J*_CH_ 164–168 Hz (N–CH=) in the ^13^C NMR spectra of these compounds can testify that the carbon atom is bound with the electronegative nitrogen atom of the amino group. The values of coupling constant ^2^*J*_CP_ 35–40 Hz indicate the *trans*-(PN)-configuration (*E*-isomer) of hexachlorophosphorates of *N*,*N*-diorganylamino-1-chlorethenyl-phosphonium (**4**–**6**) and dichloroanhydride of *N*,*N*-diorganylamino-1-chloroethenylphosphonous acids (**11**–**13**). A similar pattern (regularity) was observed in the phosphorylation products of *N*-vinylazoles and a number of other enamines [28]. A low value of ^3^*J*_PH_ (11–26 Hz) (Table 1) indicates the *cis*-orientation of the phosphorus atom relative to the vinyl proton. In the case of the *trans*-orientation, ^3^*J*_PH_ values reach 54–68 Hz [28].

**Scheme 3:**
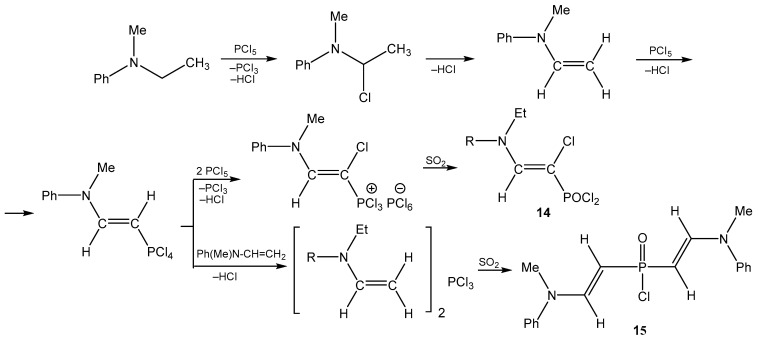
Formation of phosphorylated chloramine (**14**) and chloranhydride of bis-[2(*N*-methyl,*N*-phenylamino)ethyl] phosphonous acid (**15**).

The phosphorylation of *N*-methyl-*N*-ethylamine leads to the substitution of two chlorine atoms by the alkenyl groups in the PCl_5_ molecule to form a derivative of enaminophosphonium acids. Along with the reaction route leading to phosphorylated chloramine (**14**), the second direction is realized to afford chloroanhydride of bis-[2(*N*-methyl-*N*-phenylamino)ethyl] phosphonous acids (**15**) (Scheme 3).

In the ^31^P NMR spectrum, besides the doublet signal at δ 34.2 ppm with the value ^3^*J*_PH_ = 11.8 Hz characteristic of chloranhydrides of 2-diorganylamino-1-chlorethenyl-phosphonous acid and related to compound **14**, a signal at 35.6 ppm is present (Table 2). The splitting of this signal in a triplet of triplets unequivocally indicates the binding of the phosphorus atom with two vinyl groups in compound **15**. NMR spectral data of oxaphosphinine (**16**) are given for comparison (Table 2). The chlorophosphoryl group (POCl) in **16** also is bound to two vinyl groups, but because of the heterocyclic nature of compound **16**, vinyl protons are oriented in *cis*-position towards each other [29]. Coupling constant values ^3^*J*_PH_ for compound **16** exceed twice those of compound **15,** which correlates well with various geometry of structural fragments of these molecules containing a chlorophosphoryl group and vinyl proton. Similar conclusions follow from the comparison of ^3^*J*_HH_ values for compounds **15** and **16** (12 Hz and 6.4 Hz, respectively).

**Scheme 4:**
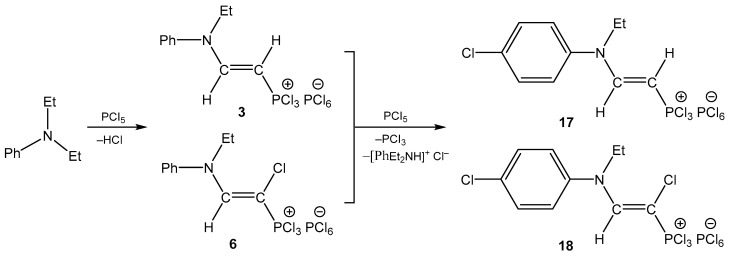
The phosphorylation of *N*,*N*-diethylaniline with phosphorus pentachloride.

The phosphorylation of *N*,*N*-diethylaniline in tetrachloromethane is accompanied (apart from phosphorylation) by the chlorination of organophosphorus compounds into the ring. In the ^31^P NMR spectrum of organyltrichlorophosphonium cations, four signals are observed. A signal at 79.8 ppm (dd) with ^2^*J*_PH_ 34.3 and ^3^*J*_PH_ 21.4 Hz is assigned to compound **3**. Apparently, a high-frequency signal of smaller intensity (82.7 ppm), also split into a doublet of doublets with very close constants ^2^*J*_PH_ 33.6 and ^3^*J*_PH_ 22.9 Hz, belongs to compound **17** (Scheme 4). Chlorination of the ethyl group in hexachlorophosphorate (**3**) is improbable, as the nitrogen atom is bound to the acceptor ethyltrichlorophosphonium group. Electrophilic hydrogen substitution into the *para*-position of the phenyl ring is more likely because it is sterically less shielded. 

Two doublets of different intensities are observed in the high field of the spectrum. The major signal at δ 72.0 ppm with ^3^*J*_PH_ = 13.7 Hz is attributed to compound **6** and completely coincides with the characteristics of hexachlorophosphorate **6**, which has been synthesized in benzene (Table 1). The minor doublet at δ 74.0 ppm with ^3^*J*_PH_ = 14.5 Hz is assigned to compound **18**. These parameters of the ^31^P NMR spectrum indicate close similarity of compounds **6** and **18**. 

## 3. The Structure of the Reaction Products of Triethylamine with Organyltrichlorophosphonium Hexachlorophosphorates

**Scheme 5:**
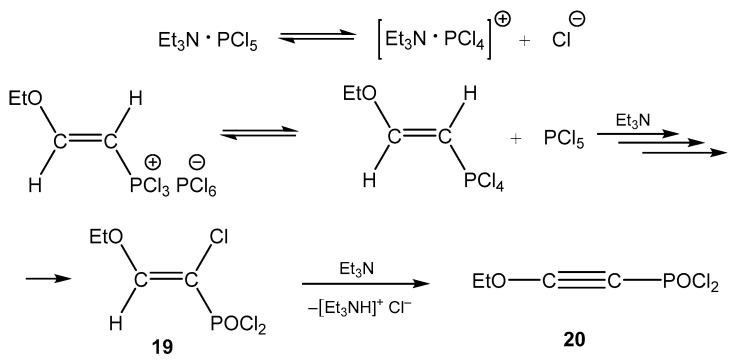
The formation of 1-chloro-2-ethoxyethenylphosphonyl dichloride (**19**) and 2-ethoxyethynylphosphonyl chloride (**20**).

The most abundant phosphorylating agents are alkyl-, aryl- and tetrachloro(styryl)phosphoranes [14,30]. The interaction of triethylamine with organic derivatives of phosphorus pentachloride, organyltrichlorophosphonium hexachlorophosphorates, in particular, 2-ethoxyethenyltrichlorophosphonium hexachlorophosphorate, has been studied by NMR spectroscopy [31]. The doublet signal at 40.4 ppm (^3^*J*_PH_ = 18.3 Hz) in the ^31^P NMR spectrum refers to the phosphorus atom in 1-chloro-2-ethoxyethenylphosphonyl dichloride (**19**). The appearance of a doublet at 36–40 ppm in the ^31^P NMR spectrum instead of a doublet of doublets is unexpected. Evidently, one vinyl hydrogen atom is replaced by the chlorine atom. The chlorination of the double bond followed by dehydrochlorination in the presence of triethylamine indicates the high electrophilicity of the chlorine atom in the donor–acceptor complexes formed between phosphorus pentachloride and triethylamine. It is most likely that these complexes are able to dissociate partially to ions (Scheme 5). The chlorine atom in the formed cation is electrophilic enough for the chlorination of a multiple bond. 1-Chloro-2-ethoxyethenylphosphonyl dichloride (**19**) is formed, most likely, according to Scheme 5.

The appearance of the dark orange color of the reaction mixture can be due to the partial dehydrochlorination of compound **19** with triethylamine to 2-ethoxyethynylphosphonyl chloride (**20**), which is unstable and undergoes easy resinification (Scheme 5) (Table 3). The intense singlet (dichlorophosphoryl substituent bound to the C≡C group) is observed at 6 ppm in the ^31^P NMR spectrum. This is consistent with the data of [32]. The ^31^P NMR spectrum also contains four doublets with pairwise coinciding coupling constants *J*_PP_ observed in the ^31^P NMR spectrum of a mixture of the products of the reaction of trichloro(2-ethoxyethenyl)phosphonium hexachlorophosphorate with triethylamine. These data indicate that the reaction affords two compounds, each of which contains two magnetically nonequivalent phosphorus atoms in the P=O groups. The doublets at 28.3 and 21.4 ppm (^2^*J*_PP_ = 56.5 Hz) belong to compound **21**, while the other compound, **22**, is characterized by the doublets at 25.6 and 21.9 ppm (^2^*J*_PP_ = 48.8 Hz) (Table 3).

**Scheme 6:**
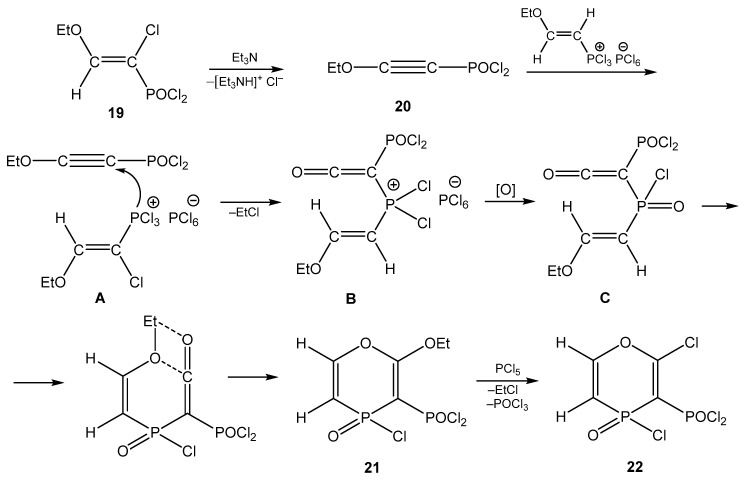
The formation of 1,4-oxaphosphinines **21** and **22**.

The spectral pattern of the obtained mixture of the reaction products changes over time. The concentration of 1-chloro-2-ethoxyethenylphosphonyl dichloride (**19**) decreases in the mixture, and the amount of two compounds (each containing two phosphorus atoms) noticeably increases. The signals at 6 ppm disappear from the spectrum. These spectral data show that compound **19** and phosphorus-containing acetylene **20** participate in the formation of the compounds containing two phosphorus atoms in the molecules. Since compound **19** is stable in the absence of triethylamine, we assume that triethylamine is also involved in the formation of diphosphorylated compounds. Based on these data and ^1^H and ^13^C NMR results, we propose the scheme for the synthesis of diphosphorylated heterocyclic compounds **21** and **22**, i.e., 1,4-oxaphosphinines containing the dichlorophosphoryl substituent in position 2 (Scheme 6).

The formation of 1,4-oxaphosphinine **21** starts from the conversion of 1-chloro-2-ethoxyethenylphosphonyl dichloride (**19**) with triethylamine to phosphorus-containing acetylene, 2-ethoxyethynylphosphonyl dichloride (**20**). Then, the attack of the trichlorophosphonium group of trichloro(2-ethoxyethenyl)phosphonium hexachlorophosphorate on the triple bond of compound **20** leads to diphosphorylated ketene **A**. The dichlorophosphonium group of diphosphorylated ketene **A** is transformed into the —POCl— group (ketene **B**) due to the phosphorylated derivatives of ethyl vinyl ether that are present in the reaction mixture. Then, heterocyclization occurs, preceded by the intramolecular *E*,*Z*-isomerization of the ethoxyvinyl fragment of the diphosphorylated ketene molecule (**B**,**C**). The formed oxaphosphinine **21** is acetal; hence, a portion of this product is transformed into 2-chlorosubstituted oxaphosphinine **22** in the presence of phosphorus pentachloride. Heterocyclization permits the rationalization of the rather high coupling constants ^3^*J*_P1H_ = 57.2 Hz for compound **21** and 62.0 Hz for compound **22**. The high values of coupling constant ^4^*J*_P2H_ = 40.4 Hz for compound **21** and 36.6 Hz for compound **22** can be explained by the W-shaped configuration of the fragment of the oxaphosphinine molecule binding the P_2_ phosphorus atom to the vinyl proton (Table 3).

The ^31^P NMR chemical shift values of the tetracoordinated phosphorus atom of POCl_2_ in compounds **19** and **20** differ several times: 80.1 and 6.0 ppm, respectively (Table 3). The presence of a triple bond (**20**) has a great shielding influence on the position of the resonance signal of phosphorus in the ^31^P NMR spectra. 

The ^13^C NMR spectra (with proton decoupling) of a mixture of compounds **21** and **22** exhibit two groups of signals characterizing the C_1_ and C_2_ atoms directly bound to the phosphorus atoms. Two doublets of doublets at 106.4–108.7 ppm shifted relatively to each other by 8.5 Hz relate to the C_1_ atoms in compounds **21** and **22**. The high values of coupling constants ^1^*J*_P1C1_ = 157.7 and 150.0 Hz and ^1^*J*_P2C1_ = 67.5 and 68.0 Hz indicate that two different phosphorus atoms are directly bonded to the C-1 carbon atom. The significant value of ^1^*J*_P1C2_ = 101.2 and 100.0 Hz in compounds **21** and **22** indicates that one phosphorus atom is bonded to the C-2 carbon atom. Thus, the ^13^C NMR spectral data are consistent with the structures of oxaphosphinines **21** and **22**. 

**Scheme 7:**
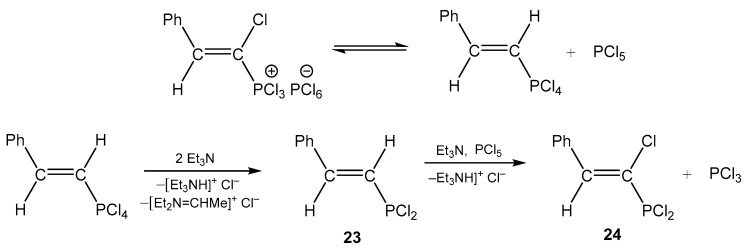
The formation of dichlorophosphines **23** and **24**.

Trichloro(styryl)phosphonium hexachlorophosphorate was used as such a model compound (Scheme 7).

Its reaction with triethylamine is accompanied by a weak coloration of the reaction mixture. The ^31^P NMR spectrum exhibits a singlet at 219.6 ppm (PCl_3_). A specific feature of the spectrum is the presence of two intense signals in the range characteristic of organic chloride of trivalent phosphorus. It is most likely that styrylphosphonyl (**23**) and 1-chloro-2-phenylethynylphosphonyl (**24**) dichlorides are formed (Scheme 7). Compound **23** is characterized by a doublet of doublets at 162.8 ppm (^2^*J*_PH_ = 9.2 Hz, ^3^*J*_PH_ = 19.1 Hz), and a doublet at 157.8 ppm (^3^*J*_PH_ = 22.1 Hz) belongs to compound **24**. A reason for the formation of compound **24** is the chlorination at the multiple bond. It should be mentioned that compound **24** was obtained [30] by heating styryltetrachlorophosphorane under much harsher conditions. The replacement of the oxygen-containing ethoxy group in hexachlorophosphorate by the phenyl group containing no oxygen atoms results in the absence of styrylphosphonyl chloride in the reaction mixture. Thus, the ^31^P NMR chemical shifts for PCl_2_ in compounds **23** and **24** in the region of 160 ppm are characteristic of a tricoordinated (trivalent) phosphorus atom. 

Thus, the NMR studies of chlorophosphorylated enamines on the basis of accessible tertiary amines and a wide range of other nitrogen-containing organic nucleophiles contributes essentially to the chemistry of unsaturated organophosphorus compounds. ^31^P NMR spectroscopy provides the most reliable and unambiguous method for the investigation of *E*–*Z*-isomeric structures of phosphorylated enamines. 

## 4. The Structural Aspects of the Phosphorylation Products of Enamides

Phosphorus-containing enamines and enamides attract the attention of researchers due to their synthetic potential and biological properties. Organophosphorus complexes are widely used in biological, pharmaceutical and material fields. Great demands on their diversity and availability encourage chemists to intensify research efforts to find effective and general routes to the synthesis of their derivatives and to study the structure of the reaction products [33,34,35,36,37]. Moreover, various enamides are suitable reactants and show high reactivity. 

**Scheme 8:**
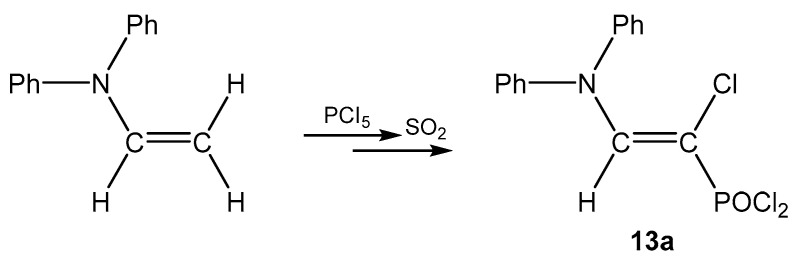
The formation of 2-diphenylaminoethene-1chloro-1-phosphonic acid dihydroanhydride (**13a**).

The low stability of alkyl- and aryl-substituted tertiary enamines in acidic media hinders the application of phosphorus pentachloride as a phosphorylating agent. So, *N*, *N*-diphenylethenamine was reacted with PCl_5_ in benzene with instant resinification, and in the reaction mixture after its treatment with sulfurous anhydride, only ^31^P NMR was used to identify 2-diphenylaminoethene-1chloro-1-phosphonic acid dichloride—*E*-(PN) isomer (Scheme 8) (see footnote in Table 1).

Enamines, containing strong electron-withdrawing substituents at the nitrogen atom and having a less nucleophilic character, behave in the phosphorylation reaction like vinyl ethers; therefore, it is possible to obtain organophosphorus compounds not contaminated with by-products in high yield.

**Scheme 9:**
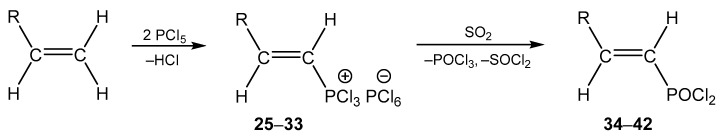
The phosphorylated enamides **25**–**42**.

The phosphorylation of *N*-vinyl-substituted tertiary amides, in which nitrogen nucleophilicity is significantly weakened, has been studied [14,38,39,40] (Scheme 9, Table 4). 

Tertiary enamides can be attacked by phosphorus pentachloride at two reaction centers, namely at the double bond of the vinyl fragment and at the carbonyl group, and the reactivity of the carbonyl group should increase upon passing from *N*-aryl-substituted to *N*-alkyl-substituted enamides.

It was found [39,40] that the studied enamides react with phosphorus pentachloride in the same way as with alkenes, i.e., at the vinyl group. Hexachlorophosphates **25**–**33** are isolated as finely crystalline precipitates that are easily hydrolyzed in air. The *N*-benzyl derivative of hexachlorophosphorate PhCH_2_N(COMe)CH=CH-${\mathrm{PCl}}_{3}^{+}$ ${\mathrm{PCl}}_{6}^{-}$ (**33a**) is formed as a slowly crystallizing oil. 

Phosphorylation products of the most reactive *N*-vinyl-*N*-alkylacetamides undergo such a rapid transformation with the participation of the carbonyl group that compounds of the **25**–**33** type cannot be detected. The replacement of the alkyl substituent at the nitrogen atom by the more electron-withdrawing benzyl group somewhat slows down the attack of phosphorus on carbonyl oxygen, and compound **33a** is stable at room temperature for 1 h. Aryl substituents at nitrogen, competing for its lone electron pair with a phosphorus-containing double bond and a carbonyl group, further reduce the nucleophilicity of the latter. This leads to a significant increase in the stability of compounds **25**–**29**. Phosphorylation products of *N*-vinyl lactams and *N*-vinylimides are stable for several days, with compound **33** being the most stable, apparently due to efficient conjugation in the condensed phthalimide system.

The ^3^*J*_HH_ value (15–16 Hz) of vinyl protons for the studied compounds **25**–**42** indicates *E*-(PN)-isomers; i.e., when going from hexachlorophosphates (**25**–**33**) to the corresponding enaminophosphoric acid dichlorides (**34**–**42**), the configuration of the molecule does not change. The ^31^P values of chemical shifts for compounds **34**–**42** are typical for acid chlorides of alkenylphosphonic acids and vary in the region of 33–37 ppm. 

**Scheme 10:**
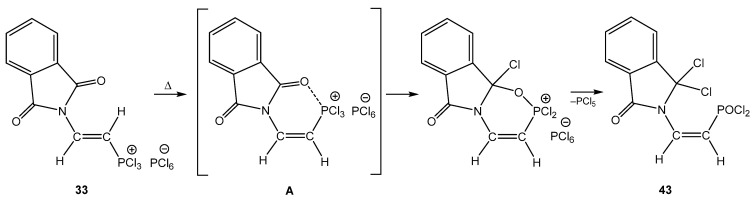
The formation of 2-(*N*-3,3-dichloro-1-hydroxyisoindolinyl)-ethenephosphonic acid dichloride (**43**).

The values of the constants ^3^*J*_PH_ = 25–27 and ^3^*J*_PH_ = 21–25 Hz are typical for *E*-isomers, for hexachlorophosphates (**34**–**42**) and for acid dichlorides (**34**–**42**), respectively. Compounds **34**–**42** are more stable than complex compounds **25**–**33**. The most stable are 2-(*N*-phenyl-*N*-benzoylamido)ethene-1-phosphonic acid dichloride (**35**) and 2-(*N*-phthalimido)ethene-1-phosphonic acid dichloride (**42**). 2-(*N*-phthalimido)ethene-1-trichlorophosphonium hexachlorophosphate (**33**) is stable at room temperature and under weak heating; at 100°C, it turns into 2-(*N*-3,3-dichloro-1-hydroxyisoindolinyl)-ethenephosphonic acid dichloride (**43**) (Scheme 10) [14,40].

The intramolecular mechanism of the formation of the phosphoryl group is evidenced by the *Z*-configuration of dichloride **43**, which inevitably arises during the intramolecular electrophilic attack of the dichlorophosphonium cation at one of the carbonyl oxygen atoms of compound **33**. The *trans-cis*-isomerization of the enaminotrichlorophosphonium group, which is necessary for the formation of the transition state (**A**), occurs either under the catalytic effect of hydrogen chloride present in the reaction medium or due to high-temperature rotation around the multiple bond without opening it. At a temperature above 100 °C, the partial resinification of the reaction mixture occurs, and the amount of chlorination products and various organophosphorus compounds of unknown structure, resulting from thermal degradation, increases. 

The ^1^H, ^13^C and ^31^P NMR chemical shifts and coupling constants of phosphonic dichloride **43** are presented in Table 5. The ^31^P NMR spectrum of compound **43** contains a single signal at 23.3 ppm in the form of a doublet of doublets. The *Z*-geometry of **43** follows from the values of the constants ^3^*J*_HH_ = 10.5 and ^3^*J*_PH_ = 57.1 Hz. A small value of the constant ^2^*J*_PC_ = 8.5 Hz also corresponds to the *Z*-(PN)-configuration. The ^35^Cl NQR spectrum of acid dichloride **43** consists of four signals (ν^77^ 26.030, 26.766, 38.072, 38.228 MHz). Two low-frequency signals refer to chlorine atoms in the POCl_2_ group since they are in the frequency range characteristic of compounds of the RPOCl_2_ series. The other two signals belong to the chlorine atoms of the CCl_2_ group (Table 5) [40].

The quantitative transition of the *E*-(PN)-isomer of hexachlorophosphate (**33**) to the *Z*-(PN)-isomer **43**, as well as the stability of the latter even at 100 °C, is unusual for the chemistry of phosphorus-containing enamines [39,41].

**Scheme 11:**
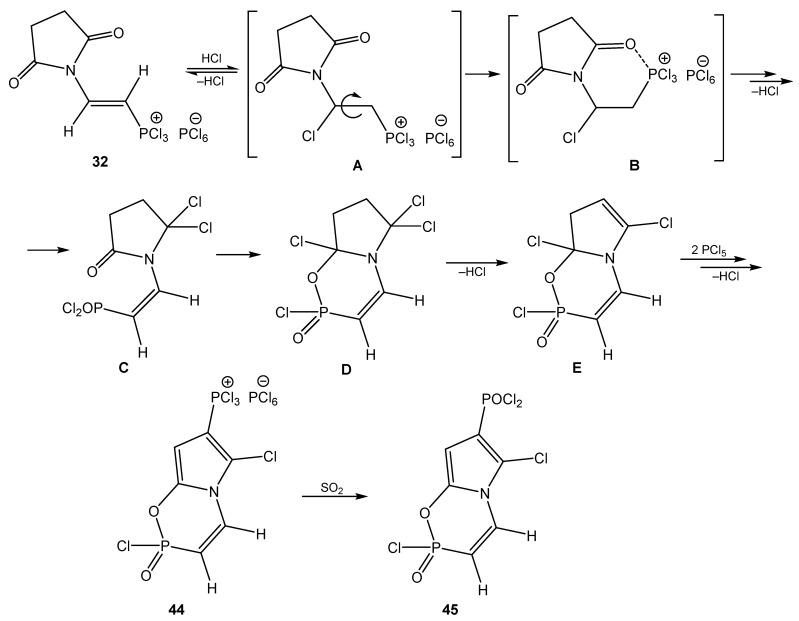
The formation of hexachlorophosphorate **44** and oxazaphosphorine **45**.

2-(*N*-succinimido)ethenyltrichlorophosphonium hexachlorophosphorate (**32**), in contrast to hexachlorophosphorate **33**, is already unstable at room temperature and gradually undergoes deep transformations, especially in the presence of phosphorus pentachloride, culminating in the formation of a bicyclic heterocycle: 2-oxo-2,6-dichloropyrrolo[1,2-e]1,5,2-oxazaphosphorin-7-trichlorophosphonium (**44**) (Scheme 11) [14,41].

The *E*,*Z*-isomerization appears to be catalyzed by the hydrogen chloride present in the reaction medium. Intermediates **A** and **B** cannot be fixed in the reaction medium due to their rapid conversion to the *Z*-isomer of 2-(*N*-5,5-dichloropyrrolidone)ethenephosphonic acid dichloride **C**. The fixation of the geometry of intermediate **C** with the maximum distance of the dichlorophosphoryl group from the dichloromethylene group is due to steric factors; i.e., general regularity of intramolecular transformations of phosphorylated enimides containing phthalimide and succinimide groups is observed. Intermediate **C** was not isolated individually, but the signal at 21.6 ppm (dd) with ^3^*J*_PH_ = 57.1 and ^2^*J*_PH _= 26.2 Hz in the ^31^P NMR spectrum corresponds to this compound. The values of the chemical shift and the coupling constants are close to those of dichloride **43** formed upon heating **33** (Scheme 10). The intermediate **C** undergoes rapid heterocyclization to bicyclic heterocycle **D**, followed by the elimination of hydrogen chloride and the formation of heterocyclic dienamine **E**. For intermediates **D** and **E**, the ^31^P NMR spectra show close values of chemical shifts of phosphorus atoms and coupling constants: 13.6 ppm (dd) with ^3^*J*_PH_ = 20.0, ^2^*J*_PH_ = 42.0 Hz, and 13.0 ppm (dd) with ^3^*J*_PH_ = 22.0, ^2^*J*_PH_ = 40.0 Hz, respectively. The compound **E** is phosphorylated at the double bond of the Δ_2_–pyrroline ring with excess phosphorus pentachloride to form 2-oxo-2,6-dichloropyrrolo[1,2-e]1,5,2-oxazaphosphorine-7-trichlorophosphonium hexachlorophosphorate (**44**). The ^31^P NMR spectrum of hexachlorophosphorate (**44**) shows three signals: 83.5 ppm, dd (${\mathrm{PCl}}_{3}^{+}$); 11.1 ppm, ddd (POCl); and a singlet of −297.7 ppm (${\mathrm{PCl}}_{6}^{-}$) (Table 5). In the ^1^H NMR spectrum, doublets of doublets at 6.44 and 7.78 ppm with proton–proton constant ^3^*J*_HH_ = 10.0 Hz correspond to vinyl protons, which indicates the *cis*-orientation of vinyl protons.

The action of SO_2_ on compound **44** gave 2-oxo-2,6-dichloro-7-dichlorophosphorylpyrrolo[1,2-e]1,5,2-oxazaphosphorine (**45**). Heterocycles **44** and **45**, despite the presence of a chloropyrrole fragment in the molecule, are stable in the absence of moisture. Two signals are observed in the ^31^P NMR spectrum of oxazaphosphorine **45**. The signal at 18.6 ppm corresponds to the exocyclic phosphorus atom, and the signal at 10.4 ppm belongs to the P(O)Cl group. The *cis*-configuration of the *N*-vinylchlorophosphoryl group is confirmed by the constants ^3^*J*_HH_ = 10.5 and ^3^*J*_PH_ = 40.5 Hz. It follows from the ^13^C NMR spectrum data that each phosphorus atom is bonded directly to one carbon atom. The spin–spin interaction of C-9 and C-8 carbon nuclei with the nuclei of both phosphorus atoms is consistent with the bicyclic structure of compound **45** (Table 5). 

**Scheme 12:**
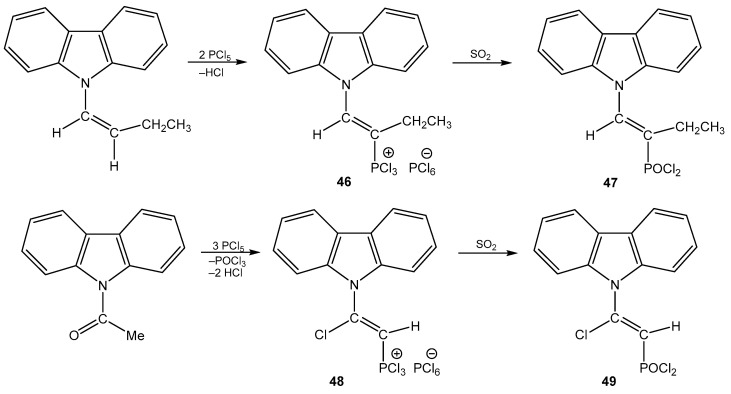
The formation of hexachlorophosphorates **46** and **48** and phosphonic dichloride **47** and **49** of *N*-substituted carbazole.

It was established [14,42] that 1-(*N*-carbazolyl)-1-butene is easily phosphorylated by phosphorus pentachloride to form 1-ethyl-2-(*N*-carbazolyl)ethene-1-trichlorophosphonium hexachlorophosphorate (**46**) and 1-ethyldichloride-2-(*N*-carbazolyl)ethene-1-phosphonic acid (**47**) (Scheme 12). In the ^31^P NMR spectrum of hexachlorophosphorate **46**, two signals are observed: 96.6 ppm, td (${\mathrm{PCl}}_{3}^{+}$), ^3^*J*_PH_ = 33.0 Hz (CH_2_, t), ^3^*J*_PH_ = 26.0 Hz (d) and 11.1 ppm singlet −297.7 (${\mathrm{PCl}}_{6}^{-}$). The value ^3^*J*_PH_ = 26.0 Hz clearly indicates the *E*-isomer (P,N) of the alkenyltrichlorophosphonium cation **46**. The ^31^P NMR spectrum of compound **47** shows 39.1 ppm, td (POCl_2_), ^3^*J*_PH_ = 27.0 Hz (CH_2_, t), ^3^*J*_PH_ = 18.5 Hz (=CH, d). 

*N*-Acetylcarbazole reacts with PCl_5_ (Scheme 12) with the formation of the corresponding complex salt, which, after the elimination of SO_2_, turns into 2-carbazolyl-2-chlorovinyl-1-phosphonic acid dichloride (**49**) [14,43]. The ^31^P NMR spectrum of compound **49** contains a single doublet at 22.4 ppm with ^2^*J*_PH _= 21.6 Hz.

**Scheme 13:**
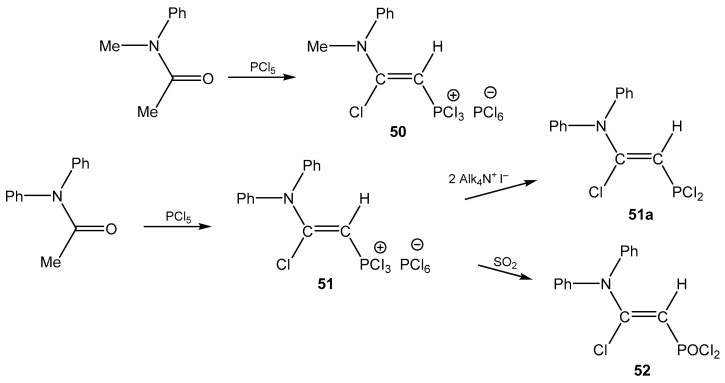
The phosphorylation of *N*-methyl-*N*-phenyl- and *N,N*-diphenylacetamides.

The attack of PCl_5_ in *N*-phenyl-substituted acetamides occurs at the oxygen of the C=O group; the intermediate unstable compound cleaves POCl_3_, turning into α-chloro-substituted tertiary enamine, which is further phosphorylated by excess phosphorus pentachloride at the carbon–carbon double bond to afford unsaturated organophosphorus compounds **50** and **51** (Scheme 13) [43,44]. Hexachlorophosphorate **51** is easily converted by the action of ${\mathrm{Alk}}_{4}N^{+}{\;I}^{\text{–}}$ into dichlorophosphine **51a**, and upon treatment with SO_2_ into phosphonic acid dichloride **52**.

The ^31^P NMR spectrum of hexachlorophosphorate **50** has an intense narrow signal of −298.1 ppm (${\mathrm{PCl}}_{6}^{-}$) and a doublet at 71.4 ppm, with ^2^*J*_PH _= 32.8 Hz (${\mathrm{PCl}}_{3}^{+}$). The ^31^P NMR spectrum of complex salt **51** contained two signals: a doublet at 75.7 ppm with ^2^*J*_PH _= 36.5 Hz, related to the organyltrichlorophosphonium cation (${\mathrm{PCl}}_{3}^{+}$), and −298.0 ppm, a broadened singlet characteristic of the ${\mathrm{PCl}}_{6}^{-}$ anion. The ^31^P NMR spectrum of compound **51a** contains two doublets, 161.9 ppm, ^2^*J*_PH_ = 2.5 Hz, and 152.9 ppm, ^2^*J*_PH_ = 4.4 Hz, in a 6:1 ratio, related to the *E*- and *Z*-isomers. In the ^1^H NMR spectrum, the doublets at 5.58 ppm with ^2^*J*_PH_ = 2.5 Hz and 6.14 ppm with ^2^*J*_PH_ = 4.4 Hz correspond to the vinyl protons of these isomers. The proton spectrum of **52** contains a multiplet in the region of 7.2 ppm (Ph) and a doublet at 5.14 ppm, ^2^*J*_PH_ = 19.5 Hz. The ^31^P NMR spectrum has a single signal at 24.6 ppm with ^2^*J*_PH_ = 19.5 Hz.

**Scheme 14:**
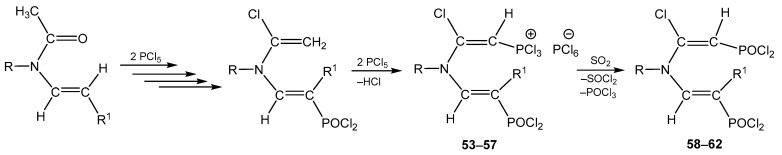
The phosphorylation of *N*-alkenylenamides.

The acetyl group of enamides of enamidotrichlorophosphonium hexachlorophosphorates, initially formed during phosphorylation of enamides, participates in further transformations of organophosphorus compounds, which involve phosphorus chloride substituents at the double bond. It is established that enamidotrichlorophosphonium hexachlorophosphorates containing an acetamide group in the molecule in the presence of PCl_5_ are rapidly converted upon heating and more slowly at room temperature into 2-*N*-alkyl(aryl)-*N*-2-(dichlorophosphoryl)-alkenylamino-2-chloroethenylphosphonium hexachlorophosphorates (**53**–**57**) (Scheme 14) [14,40,41,42,45,46,47].

The formation of compounds **53**–**57** is the result of an intramolecular attack of the carbonyl group by the organyl trichlorophosphonium oxygen cation, leading to the appearance of a vinyl chloride group, which then reacts with PCl_5_ similarly to the phosphorylation of enamines. Hexachlorophosphorates **53**–**57** cannot be isolated individually without impurities, since the reaction mixture, along with them, always contains products of further transformations. However, after treatment of compounds **53**–**57** with sulfur dioxide, stable diphosphonic acid chlorides are formed (**58**–**62**) (Table 6). 

The value of the constant ^3^*J*_HH_ = 14–16 Hz, as well as ^3^*J*_PH_ = 20–26 Hz, indicates *E*-isomerization of (P,N)-organophosphorus compounds **53**–**62**. For compounds **53**, **54**, **56**, **58** and **60**, the constants ^2^*J*_PH_ and ^3^*J*_PH_ in the ^31^P NMR spectra are almost equal in magnitude.

The ^35^Cl NQR spectrum of compound **59** consists of four signals: ν^77^ 26.608, 26.734, 26.933 and 36.775 MHz. The high-frequency signal (36.775 MHz) relates to the chlorine atom bonded to the carbon atom, and the three low-frequency signals belong to the chlorine atoms bonded to the phosphorus group POCl_2_.

## 5. The Structure of Enamide-Derived Azaphosphorines 

**Scheme 15:**
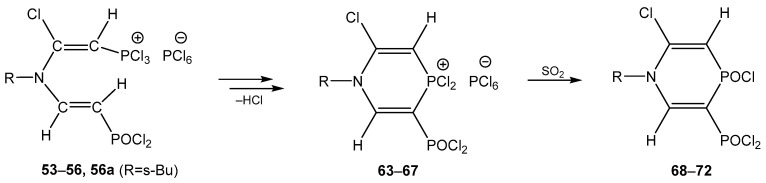
The formation of hexachlorophosphorates of 1,4-azaphosphoniarines (**63**–**67**) and 1,4-azaphosphorines (**68**–**72**).

It has been established that heating or long-term room-temperature storage of bisphosphorylated divinylamines (**53**–**56**, **56a**) delivers cyclic hexachlorophosphorates of 1,4-azaphosphoniarines (**63**–**67**). The formation of compounds **63**–**67** is the result of an intramolecular attack by the ethenyltrichlorophosphonium cation at the π-bond of the ethenyldichlorophosphoryl group. The transformation of disphosphorylated dialkenyls into heterocycles (**63**–**67**) is noteworthy in that the authors of [14,47] using the trichlorophosphonium cation managed to phosphorylate a carbon–carbon double bond having a strong electron-withdrawing substituent. Compound **57** is unable to form the 1,4-azaphosphorine ring, apparently due to steric hindrance created by three substituents at the double bond for intramolecular cation attack of 1,4-azaphosphorine derivatives (**68**–**72**) (Scheme 15) (Table 7) [46,47].

The transformation of compounds **63**–**67** under the action of SO_2_ into 1,4-azaphosphorines (**68**–**72**) occurs much more slowly than similar transformations of acyclic alkenyltrichlorophosphonium hexachlorophosphonates.

The coupling constant ^2^*J*_PH_ of the proton of the P–CH= group (${\mathrm{PCl}}_{3}^{+}$) is almost not observed in the ^31^P NMR spectra, and only for some compounds is it possible to fix the constant in the range of 0.6 Hz. The insignificant value of the geminal constant is characteristic of 1,4-heterophosphorine cycles. The unusually large coupling constant across four bonds (^4^*J*_PH_) is due to the W-shaped planar structure of the molecular fragment of these compounds, which includes both phosphorus atoms and a proton. The value of the coupling constant ^2^*J*_PP_ (15–18 Hz) is typical for geminal bonded phosphorus atoms. The existence of the C-Cl bond in the molecule of compounds **63**–**72** is confirmed by the presence of signals in the ^35^Cl NQR spectra in the frequency range of 38.7–39.4 MHz.

**Scheme 16:**
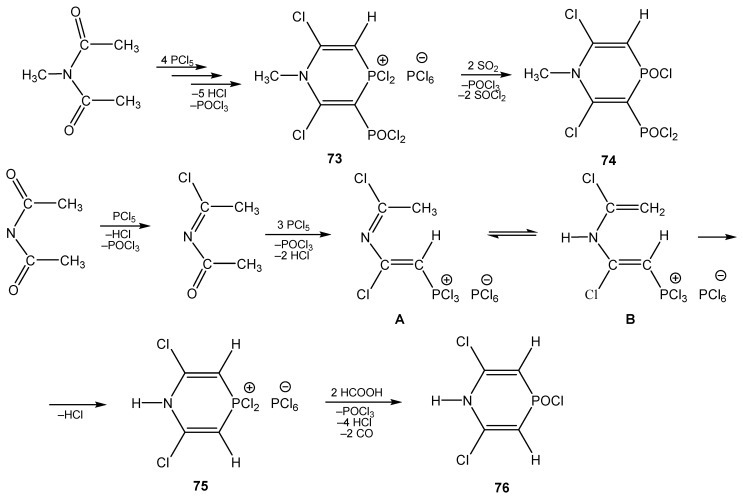
The formation of 1,4-azaphosphorinonium hexachlorophosphorates **73** and **75** and 1,4-azaphosphorines **74** and **76**.

Similarly, the reaction of *N*-methyl-*N*,*N*-diacetamide with phosphorus pentachloride leads to 1,4-azaphosphorine containing an exocyclic phosphoryl group [41,48], while the phosphorylation of *N*,*N*-diacetamide proceeds differently [49] (Scheme 16).

The reaction, carried out under moderate vacuum conditions, furnishes 1*H*-2,4,4,6-tetrachloro-1,4-dihydro-1,4-azaphosphorinonium hexachlorophosphorate (**75**). The formation of compound **75** is probably preceded by imine–enamine rearrangement, which converts imidoyl chloride **A** to dialkenylamine **B**. Intramolecular phosphorylation of the latter leads to the formation of heterocycle **75**, which is transformed by formic acid to 1*H*-4-oxo-2,4,6-trichloro-1,4-dihydro-1,4-azaphosphorine (**76**) (Table 8).

The phosphorus signal of ${\mathrm{PCl}}_{6}^{-}\;$in the ^31^P NMR spectra of compounds **73** and **75** appears in the region of −297.2 and −298.9 ppm, respectively. The large value of the direct coupling constant ^1^*J*_CH_=180.0–187.0 Hz may indicate the influence of the lone electron pair (LEP) of the oxygen atom on the C-H bond, and in addition, the proximity of the carbon atom to the electronegative phosphorus (or nitrogen) atom in the cycle [50,51,52,53]. NQR spectroscopy data also indicate the formation of heterocyclic compounds. It should be noted that ^35^Cl NQR spectroscopy is successfully used to prove the structure of chlorine-containing compounds [54].

So, diacetamide reacts with phosphorus pentachloride to give the cyclic phosphonium salt (**75**) which is converted to azaphosphorinone (**76**) upon treatment with formic acid. The ^31^P NMR chemical shift values of POCl in 1,4-oxaphosphinines **21** and **22**, azaphosphorines **68**–**72** and azaphosphorinone **76** appear in the region 24–28 ppm and practically do not depend on the nature (structure) of the heterocycle. 

**Scheme 17:**
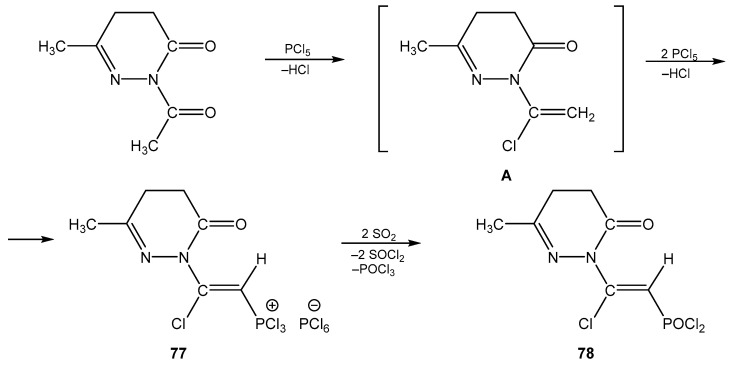
The phosphorylation of *N*-acetylhydropyridazine.

The phosphorylation of a representative of cyclic *N*-acylated hydrazones, 1-acetyl-3-methyl-6-oxo-1,4,5,6-tetrahydropyridazine, was carried out (Scheme 17) [55].

The process proceeds similarly to the phosphorylation of *N*,*N*-disubstituted acetamides [43]. The carbonyl group of the heterocycle lowers the nucleophilicity of the *N*-acetyl nitrogen atom, directing the reaction to the formation of α-chlorenamine **A**. The intermediate **A** is further phosphorylated by phosphorus pentachloride to afford complex salt **77**, from which dichloride 2-(3-methyl-6- oxo-1,4,5,6-tetrahydropyridazin-1-yl)-2-chloroethenylphosphonic acid (**78**) is easily obtained. The ^31^P NMR spectrum of dichloride **78** contains a doublet at 33.1 ppm with ^2^*J*_PH_ = 24.8 Hz.

**Scheme 18:**
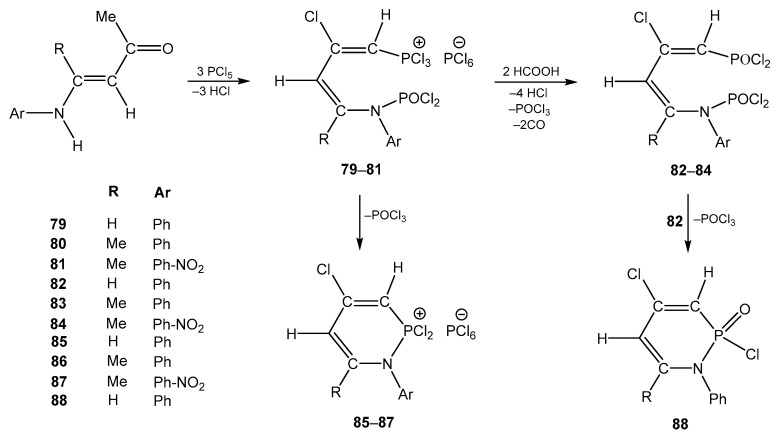
The phosphorylation of enamino ketones.

Enamino ketones as well as ethyl ethers of phenylaminocrotonic acids are convenient starting compounds for the synthesis of nitrogen–phosphorus-containing heterocycles via phosphorylation [56,57,58]. 4-Arylamino-3-penten-2-ones react with PCl_5_ to give 4-(*N*-aryl-*N′*-dichlorophosphorylamino)-2-chloro-1,3-dienyltrichlorophosphonium hexachlorophosphorates **79**–**81**. Compounds **79**–**81** and diphosphonic acid chloride **82** readily formed from **79** undergo gradual heterocyclization to 1,2-dihydro-1,2-azaphosphorines **85**–**87** and compound **88** with the elimination of phosphorus oxychloride (Scheme 18) (Table 9) [56,57].

The ^1^H, ^13^C and ^31^P NMR chemical shifts and coupling constants of phosphonic chloride **88** (CDCl_3_) are presented in Table 9. The ^31^P NMR chemical shifts of **85**, **86**, and **87** have the following values (ppm): 56.2 dd, ^3^*J*_PH _= 34.4 and ^2^*J*_PH_ = 16.5 Hz; 57.3 d, ^2^*J*_PH _= 17.8 Hz; and 60.4 d, ^2^*J*_PH _= 20.1 Hz, respectively. 

**Scheme 19:**
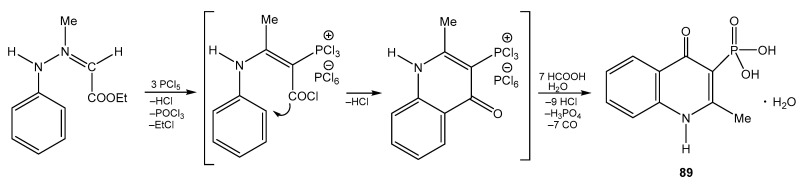
The phosphorylation of ethyl ether of phenylaminocrotonic acid.

It has been found that a longer (for several hours) treatment of ethyl phenylaminocrotonoate with phosphorus pentachloride leads to 1,4-quinolone **89** containing a trichlorophosphonium group in position 3 (Scheme 19) (Table 9) [58]. The heterocyclization is preceded by the formation of phosphorus-containing phenylaminocrotonic acid chloride. Its carbonyl group exhibits increased electrophilicity. 

We found that 2-methyl-1,4-quinolone is not phosphorylated by phosphorus pentachloride, which confirms the proposed scheme for the reaction of crotonate with PCl_5_, according to which the phosphorylation at the double bond is preceded by acylation of the benzene ring. The phosphorylation at the NH nitrogen atom of the quinolone is also possible, but the NH bond in the resulting phosphonic acid is regenerated in the course of hydrolysis.

The nitrogen chemical shift in the ^15^N NMR spectrum of compound **89** is at −220.1 ppm, which is characteristic of a pyrrole-type nitrogen atom [52,53]. 

**Scheme 20:**
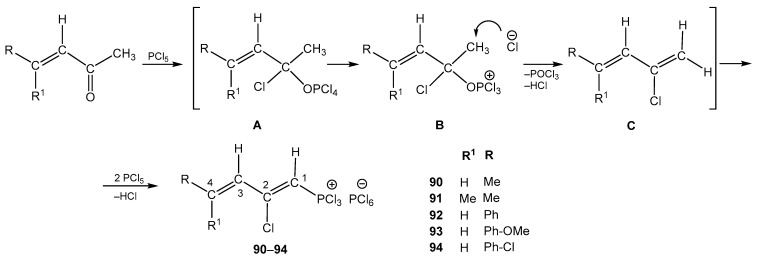
Chlorophosphorylation of unsaturated ketones.

It should be noted that unsaturated ketones behave similarly to enamino ketones when phosphorylated with phosphorus pentachloride [59,60]. *C*-phosphorylation products dienyltrichlorophosphonium hexachlorophosphorates have been studied by means of 2D and multinuclear ^1^H, ^13^C and ^31^P NMR spectroscopy. The reaction most probably starts with the attack of phosphorus pentachloride at the carbonyl group to form *O*-phosphorylated product **A** (Scheme 20) [59]. 

In the presence of excess phosphorus pentachloride, tetrachlorophosphorane **A** undergoes ionization to give intermediate **B**, which decomposes to afford diene **C**. The latter is phosphorylated by excess phosphorus pentachloride similarly to the known alkenes with the formation of hexachlorophosphorates **90**–**94**. The ^31^P NMR spectra of compounds **90**–**94** have signals at 84–89 ppm belonging to the ${\mathrm{PCl}}_{3}^{+}$ cation; a broadened signal at −297 to −298 ppm corresponds to the ${\mathrm{PCl}}_{6}^{-}$ anion (Table 10). 

The ^13^C NMR spectra of hexachlorophosphorates **90**–**94** contain doublet signals in the region of 116–119 ppm, which are characteristic of the carbon atom directly bonded to the phosphorus atom (Table 10). The coupling constant ^1^*J*_PC_ lies in the range of 115–117 Hz, but the spin–spin coupling constant ^2^*J*_PH_ between the phosphorus atom and vinyl protons turns out to be quite sensitive to the effect of substituents (41–56 Hz).

**Scheme 21:**
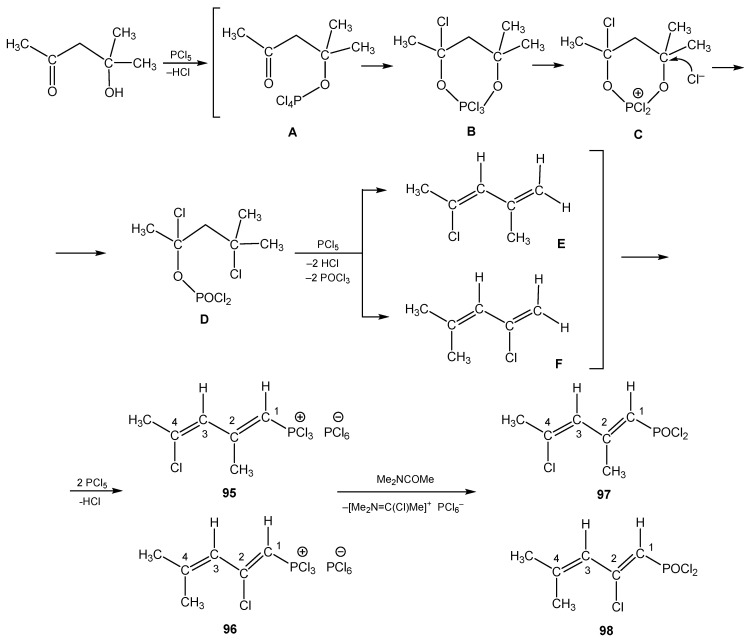
Chlorophosphorylation of diacetone alcohol.

Chlorophosphorylated 1,3-dienes are also formed by the action of PCl_5_ on diacetone alcohol [60]. It has been established that two pentadienyltrichlorophosphonium hexachlorophosphates (**95**, **96**) are produced in approximately equal amounts via phosphorylation. Under the action of dimethylacetamide, hexachlorophosphorates (**95**, **96**) are converted into a mixture of two isomers: 2-chloro-2,4-pentadiene-4-methyl-5-phosphonic (**97**) and 2-methyl-2,4-pentadiene-4-chloro- 5-phosphonic (**98**) acids (Scheme 21).

The process starts from the attack by the hydroxy oxygen atom on the phosphorus atom of phosphorus pentachloride, which is evidenced by a vigorous evolution of hydrogen chloride. The formed intermediate **A** readily undergoes cyclization to trichlorophosphorane **B** as a result of the intramolecular attack by the carbonyl oxygen atom of intermediate **A** on the phosphorus atom of tetrachlorophosphorane group. The attack by the carbonyl group of compound **A** at phosphorus pentachloride present in the reaction mixture in excess is unlikely because the intramolecular process is preferable. The intermediate **B** readily dissociates to ions, and the formed quasiphosphonium salt **C**, according to the second step of the Arbuzov reaction, is transformed to compound **D**. The subsequent conversion of **D** to dienes **E** and **F** proceeds in the presence of phosphorus pentachloride with the elimination of trichlorophosphorus oxide and hydrogen chloride, similarly to the process of the formation of alkenes in the phosphorylation of tertiary alcohols containing at least two methyl groups next to the hydroxy group with phosphorus pentachloride. The process is finalized by the phosphorylation of pentadienes with phosphorus pentachloride (Scheme 21).

The structure of the obtained products has been established by two-dimensional and multinuclear ^1^H, ^13^C and ^31^P NMR spectroscopy. In the ^31^P NMR spectra of dienyltrichlorophosphonium hexachlorophosphorates (**95**, **96**) recorded both in nitrobenzene and in nitromethane, two doublets of approximately the same intensity and a broadened singlet in the region of −296 ppm, characteristic of the ${\mathrm{PCl}}_{6}^{-}$ anion, are observed in the region 90.5 ppm (d, ^2^*J*_PH_ 52.8 Hz) and 85.7 ppm (d, ^2^*J*_PH_ 56.0 Hz). The ^13^C NMR spectrum of a mixture of compounds **95** and **96** shows two doublets at 116.5 ppm (C-1, ^1^*J*_PC_ 114.9 Hz) and at 114.8 ppm (C-1, ^1^*J*_PC_ 111.2 Hz) characteristic of carbon atoms directly bonded to the phosphorus atom. In the ^31^P NMR spectrum of a mixture of isomers **97** and **98**, signals detected in the region 26.2 ppm (d, POCl_2_, ^2^*J*_PH_^1^ 35.3 Hz) and 25.6 ppm (d, POCl_2_, ^2^*J*_PH_^1^ 36.1 Hz) relate to compounds **97** and **98**, respectively. The ^1^H NMR spectrum of compound **97** contains signals at 2.36 ppm (d, C(2)Me, ^4^*J*_PH_ 3.5 Hz) and 2.29 ppm (s, C(4)Me). The ^1^H NMR spectroscopy data of diene **97** indicate the *cis*-orientation of the methyl group towards the POCl_2_ moiety in the propenyl fragment. This is evidenced by the value of the constant ^4^*J*_PH_ = 3.5 Hz. 

**Scheme 22:**
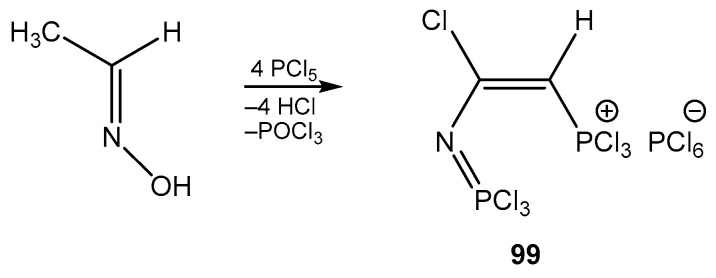
The phosphorylation of acetaldoxime.

It was established [61] that acetaldoxime reacts with PCl_5_ to form the stable phosphorus-containing enamine **99**. The trichlorophosphase group formed in compound **99** reduces the nucleophilicity of the nitrogen atom, which ensures the stability of this compound (Scheme 22).

2-(Trichlorophosphazo)-2-chloroethenyltrichlorophosphonium hexachlorophosphorate (**99**) is isolated as a white powder. The ^31^P NMR spectrum of compound **99** shows δ 80.5 ppm, d (${\mathrm{PCl}}_{3}^{+}$), ^2^*J*_PH_ = 44.2 Hz; δ 15.9 ppm, d (P=N), ^4^*J*_PH_ = 15.4 Hz; δ –297.8 ppm, br s (${\mathrm{PCl}}_{6}^{-}$). In the ^1^H NMR spectrum in the region of vinyl protons, there is a single signal at 5.67 ppm, dd, which indicates that both PCl_3_ groups belong to the same molecule.

**Scheme 23:**
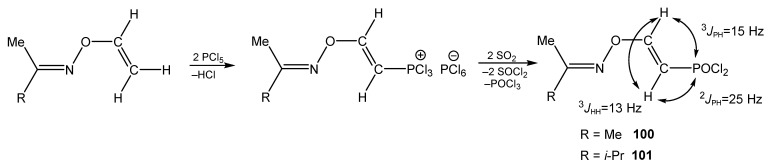
The phosphorylation of *O*-vinyl oximes.

Oxime derivatives containing a vinyl group at the oxygen atom react with PCl_5_ in the same way as vinyl esters [62]. *O*-vinyloximes are phosphorylated at the *O*-vinyl group at room temperature, forming alkenyltrichlorophosphonium hexachlorophosphorates (Scheme 23).

Under the action of sulfur dioxide, hexachlorophosphorates are converted into *E*-ethenylphosphonic acid dichlorides (**100**, **101**) (^3^*J*_HH_ = 13.0 Hz). The structure of the compounds has been established by 2D and multinuclear ^1^H, ^13^C and ^31^P NMR spectroscopy [62]. 

## 6. The Structural Features of Acetylurea-Derived Diazaphosphorines 

Organophosphorus compounds obtained by phosphorylation of urea derivatives and related compounds are widely used in the life sciences, medicine chemistry, pharmaceuticals, organic catalysis and materials science [63,64,65,66,67,68,69,70,71,72]. Efficient P-C or P-N bond formation using the various available phosphorus sources plays a critical role in the construction of organophosphorus compounds. Among them, PCl_5_ acts as an essential source of phosphorus; therefore, its use to form P-C or P-N covalent bonds is being actively developed.

The problem of obtaining phosphorylated nitrogen-containing heterocyclic compounds, which play an important role in medicine, agrochemistry and modern metal complex catalysis, deserves special attention. The need to select conditions for each particular type of heterocycles, harsh reaction conditions (high temperature, the presence of strong bases and acids) and the high cost of the catalysts used significantly limit the application of this approach in practice. An alternative devoid of these disadvantages can be the construction of a heterocyclic ring using universal phosphorus pentachloride. With a rational approach, the “multiple” reactivity of such a reagent will provide an arsenal of substrate-controlled transformations leading to structurally diverse products.

Due to the wide practical application, the chemistry of organophosphorus compounds is developing rapidly. Among numerous series, phosphorus-containing heterocycles have been synthesized, which can be represented as structural analogues of azabenzenes: pyridine, pyrimidine, *s*-triazine. The interaction of urea derivatives with phosphorus pentachloride and the phosphorylation products have been successfully studied [73,74,75,76,77,78,79,80,81,82,83,84]. It is known that the results of phosphorylation of urea derivatives depend on the conditions of the process.

**Scheme 24:**
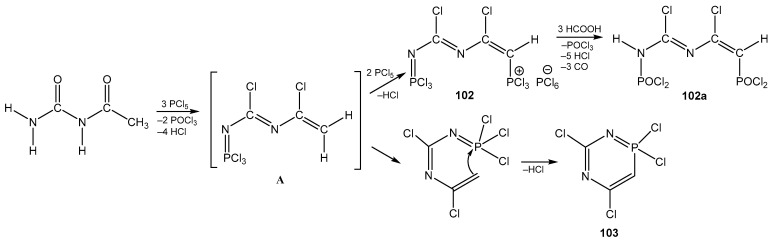
The chlorophosphorylation of *N*-acetylurea.

The direction of phosphorylation of *N*-acetylurea under the action of phosphorus pentachloride significantly depends on the ratio of reagents (Scheme 24) [73,76,77,78,79]. Further transformations of intermediate **A** are determined by the presence or absence of PCl_5_ in the reaction medium. At a molar ratio of urea and PCl_5_ of 1:5, hexachlorophosphorate **102** is obtained. However, in the absence of an excess of PCl_5_ in the reaction medium, intramolecular phosphorylation of the C=C double bond of azabutadiene **A** by the trichlorophosphazol group occurs to deliver 2,2,4,6-tetrachloro-2,2-dihydro-1,5,2-diazaphosphorine **103** (Scheme 24) (Table 11). The action of formic acid on complex compound **102** gives *N*^1^-dichlorophosphoryl-*N*^2^-(1-chloro-2-dichlorophosphorylethenyl)-C-chloroformamidine **102a** [73,79].

The ^31^P NMR spectrum of compound **102** is represented by three signals: δ, ppm, 82.8 d (${\mathrm{PCl}}_{3}^{+}$), ^2^*J*_PH_ = 43.2 Hz; 23.7 s (${\mathrm{PCl}}_{3}$); −297.2 s (${\mathrm{PCl}}_{6}^{-}$). A single signal at 6.55 ppm (=CH) is observed in the proton spectrum. The ^13^C NMR spectrum of complex compound **102** shows the following: δ, ppm, 156.00 dd (ClC=CH), ^2^*J*_PC_ = 10.2 and ^4^*J*_PC_ = 2.0 Hz; 144.12 dd (ClC=N), ^2^*J*_PC_ = 6.8 and ^4^*J*_PC_ = 2.0 Hz; 95.62 (=CH). The presence of a spin–spin interaction of each of the carbon atoms simultaneously with two phosphorus atoms proves the presence of two phosphorus atoms in one molecule.

The ^31^P NMR spectrum of chloroformamidine **102a** has two signals, δ, ppm: 25.3 d (C-POCl_2_), ^2^*J*_PH_ = 19.8 Hz, and 3.3 s (N-POCl_2_). In the ^1^H spectrum, the signal =CH of the proton is in the region of 6.2 ppm. Two high-frequency signals in the ^35^Cl NQR spectrum of compound **102a** at 36.210 and 37.243 MHz refer to the chlorine atoms of the C–Cl bond, the latter being related to the chlorine atom in the C(Cl)=CH group. Two low-frequency signals at 27.638 and 27.088 MHz refer to chlorine atoms in the POCl_2_ group.

**Scheme 25:**
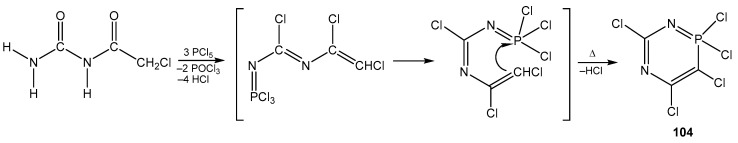
The formation of 2,2,3,4,6-pentachloro-2,2-dihydro-1,5,2-diazaphosphorine (**104**).

Heating of the reaction mixture obtained by the interaction of PCI_5_ and chloroacetylurea is accompanied by the release of hydrogen chloride and leads to the formation of 2,2,3,4,6-pentachloro-2,2-dihydro-1,5,2-diazaphosphorine (**104**) (Scheme 25) [76].

The ^31^P NMR spectrum of diazaphosphorine (**104**) contains a singlet at 44.7 ppm, located in the region characteristic of azaphosphorines containing a PCl_2_ group in the heterocycle (Table 11). The structure of heterocycle **104** is also confirmed by the data of ^35^Cl NQR spectroscopy [76]. 

**Scheme 26:**
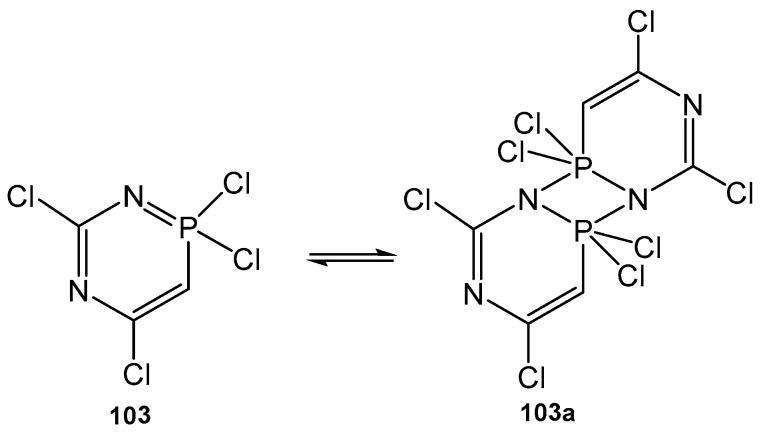
The formation of dimeric diazaphosphorine (**103a**).

Upon transition of diazaphosphorine (DAP) (**103**) to the crystalline state, the conjugation of the P=N, C=C and C=N bonds is disturbed or significantly weakened, as indicated by a significant change in the characteristic vibrations in the IR spectra of these structures [78]. The violation of conjugation occurs, most likely, as a result of the dimerization of diazaphosphorine (**103**) at the P=N bond, and the resulting dimer (**103a**) is stable only in the crystalline state, and upon melting or dissolution, it dissociates extremely easily to give the monomeric form (**103**) (Scheme 26).

In the ^31^P NMR spectra of solutions of compound **103** at room and low (−80 °C) temperatures, no signals are observed in the region characteristic of the pentacoordinated phosphorus atom, which confirms the conclusion that only the monomeric form is present in the solution. The dimeric structure of DAP (**103a**) in the crystalline state is confirmed by NQR and photoelectron spectroscopy data, as well as by quantum chemical calculations [78,85]. A weighty argument in favor of the dimeric structure (**103a**) is the significant splitting of the resonant doublet signal (ν^77^ 27.788 and 29.308 Hz), which characterizes the PCl_2_ fragment in the NQR ^35^Cl spectrum of compound **103a**. This indicates that the electronic distribution (state) of chlorine atoms differs significantly. Upon dimerization of **103** to **103a**, the phosphorus atom becomes pentacoordinated, thereby acquiring a trigonal bipyramidal configuration. As a result, the nitrogen atoms of the four-membered cycle can occupy axial (one) and equatorial (second) positions. Accordingly, one chlorine atom becomes axial, and the other becomes equatorial, which causes the observed splitting of the ν^77^ (P-Cl) signal. In the NQR ^35^Cl spectra of chlorophosphoranes, the signals of chlorine nuclei occupying the equatorial position in the trigonal bipyramid are observed at higher frequencies than the signals of axial chlorine atoms.

**Scheme 27:**
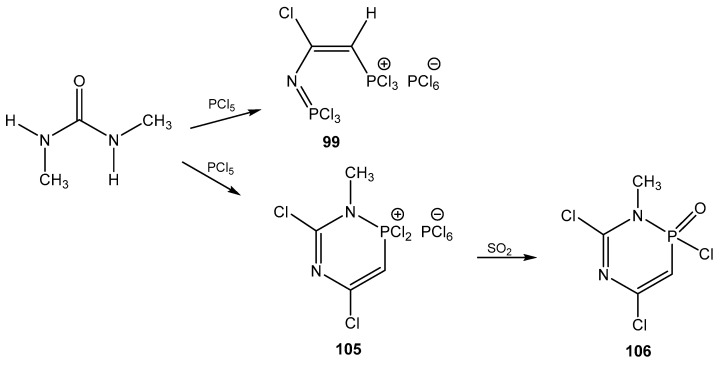
The formation of diazaphosphorines **105** and **106**.

The interaction of PCl_5_ and *N*-methyl-*N*′-acetylurea unexpectedly affords 2-(trichlorophosphazo)-2-chloroethenyltrichlorophosphonium hexachlorophosphorate (**99**) (see Scheme 22), and a minor amount of 1-methyl-2,2,4,6-tetrachloro-1,2-dihydro-1,5,2-diazaphosphorinonium hexachlorophosphorate (**105**) is formed (Scheme 27). Under the action of SO_2_, compound **105** is readily converted to 1-methyl-2,4,6-trichloro-1,2-dihydro-1,5,2-diazaphosphorine (**106**) (Table 11). Both heterocycles are easily hydrolyzable crystals, stable without moisture access [14,75].

The structures of compound **99** and heterocycles **105** and **106** were proved by ^1^H, ^13^C and ^31^P NMR methods, as well as ^35^Cl NQR spectroscopy data. The ^31^P NMR spectrum of compound **105** has a doublet at 64.4 ppm corresponding to the phosphorus atom of the PCl_2_ group and a broadened singlet in the region of −297.5 ppm (Table 11). 

**Scheme 28:**
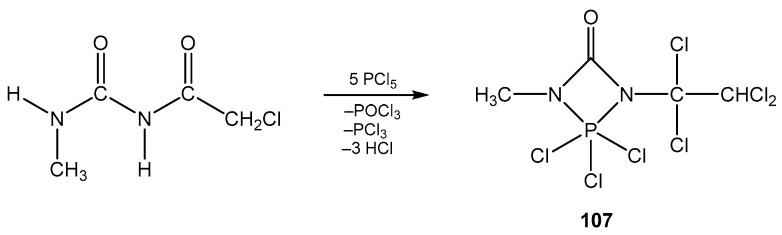
The formation of phosphetidinone **107**.

Under the action of PCl_5_ on *N*-methyl-*N*′-chloroacetylurea, the chloroacetyl group is not phosphorylated; the reaction furnishes a compound with a four-membered diazaphosphetidine ring—1-methyl-2,2,2-trichloro-3-(1,1,2,2-tetrachloroethyl)-1,3-diaza-2-phosphetidinone (**107**) (Scheme 28) [84].

The structure of compound **107** has been established by NMR spectroscopy data. In the ^31^P NMR spectrum of phosphetidinone **107**, a single signal refers to the pentacoordinated phosphorus atom: −69.1 ppm, qd, ^3^*J*_PH_ = 24.4 and ^4^*J*_PH_ = 1.0 Hz (Table 11).

**Scheme 29:**
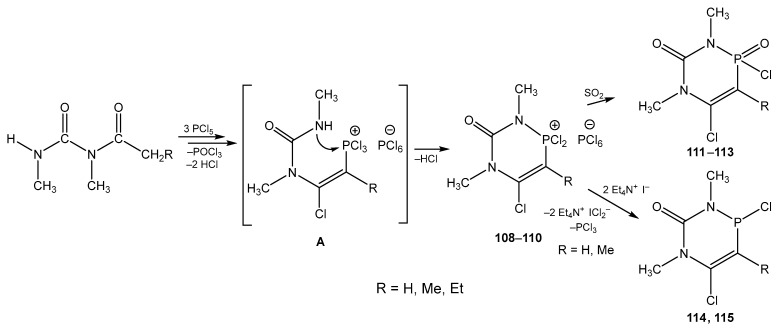
The formation of six-membered heterocycles **108**–**115**.

The phosphorylation of *N*-acyl-*N*,*N*′-dimethylurea derivatives with phosphorus pentachloride gives rise to six-membered heterocycles, 1,5-dimethyl-2,2,4-trichloro-6-oxo-3-alkyl-1,5,2-diazaphosphoniarin hexachlorophosphorates (**108**–**110**) (Scheme 29) [74].

The phosphorylation begins with the attack of phosphorus pentachloride at the oxygen atom of the acetyl group to produce α-chloramine. The enurea is further phosphorylated at the double bond with the formation of acyclic hexachlorophosphorate 2-(*N*,*N*′-dimethylurea)-2-chloro-1-alkylethenyltrichlorophosphonium **A**. The subsequent rapid intramolecular attack of the chlorophosphonium cation at the secondary nitrogen atom of urea ends with cyclization to deliver diazaphosphorines (**108**–**110**). The action of SO_2_ on compounds **108**–**110** results in 1,5-dimethyl-2,4-dichloro-2,6-dioxo-3-alkyl-1,2,5,6-tetrahydro-1,5,2-diazaphosphoniarins (**111**–**113**) (Table 12). 

The signals of the tetracoordinated phosphorus atom of the ${\mathrm{PCl}}_{2}^{+}$ group in compounds **63**–**67** (Table 8), **73** and **75** (Table 8), **85**–**87** and **105** (Table 11), and **108**–**110** (Table 12) in the ^31^P NMR spectra are observed in a higher frequency field (45–64 ppm) than those of the POCl moiety in products **111**–**113** (17–19 ppm) (Table 12), which is apparently more associated with the existence of a molecule in a charged form. 

Reduction of complex compounds **108**–**110** with tetraethylammonium iodide gives 1,5-dimethyl-2,4-dichloro-6-oxo-3-alkyl-1,2,5,6-tetrahydro-1,5,2-diazaphosphoniarines (**114**, **115**) (Scheme 29) (Table 12) [74]. In the ^31^P NMR spectrum of compound **115**, a multiplet signal is observed in the region of 99.3 ppm, which corresponds to the phosphorus atom of the PCl group. It should be noted that the ^31^P NMR chemical shift of the tricoordinated (trivalent) phosphorus atom of the PCl group (**114**, **115**) is quite large, as expected, and lies in the region of approximately 100 ppm (Table 12).

**Scheme 30:**
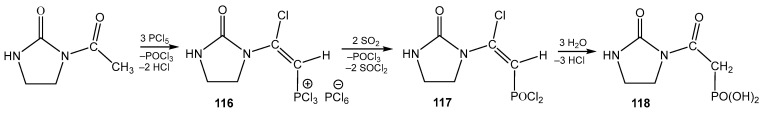
The chlorophosphorylation of 1-acetylimidazolidin-2-one.

*N*-acetyl-*N*,*N*′-ethyleneurea (1-acetylimidazolidin-2-one) reacts with PCl_5_ to form 2-(2-oxo-1-imidazolidinyl)-2-chloroethenyltrichlorophosphonium hexachlorophosphorate (**116**) (Scheme 30) [81].

Phosphorus pentachloride attacks the oxygen atom of the acetyl group of acetylethyleneurea, but steric and conformational hindrances in cyclic ureide do not allow intramolecular attack of the nitrogen atom N-H by the organyltrichlorophosphonium cation (**116**) with the formation of a heterocycle, as observed in the phosphorylation of acyclic trisubstituted ureides [74]. Compound **116**, upon treatment with SO_2_, is converted into 2-(2-oxo-1-imidazolidinyl)-2-chloroethenylphosphonic dichloride (**117**); its hydrolysis yields 2-oxo-2-(2-oxo-1-imidazolidinyl) ethylphosphonic acid (**118**) (Table 13). 

The ^31^P NMR chemical shifts of **116** have the following values (ppm): 80.2 d, ^2^*J*_PH _= 38.9 Hz (${\mathrm{PCl}}_{3}^{+}$) and −295.5 br s (${\mathrm{PCl}}_{6}^{-}$).

A generalization of the analysis of ^31^P NMR chemical shifts of nitrogen–phosphorus-containing enamines and the corresponding heterocycles shows that a change in the coordination number of the phosphorus atom from 3 to 6 significantly changes δ^31^P from about + 200 to −300 ppm. As regards the spin–spin coupling constants, the Karplus equation is applicable to the saturated systems of the H–C(*sp*^3^)–C(*sp*^3^)–H type. The vicinal interaction in such systems strongly (more precisely, mainly) depends on the value of the dihedral angle φ. If one of the carbon atoms is in the *sp*^2^ hybridization state, the Karplus equation becomes inapplicable. In our case, we deal with the unsaturated compounds (enamines), in which carbon atoms are in the state of *sp*^2^ hybridization. 

## 7. Conclusions

The reactions of phosphorus pentachloride with enamines, tertiary amines, enamides, diacetamides, *N*-vinyl-substituted cyclic imides and *N*-acetylureas can be employed as a platform for the simple and convenient synthesis of hard-to-reach nitrogen-containing organophosphorus compounds and new types of heterocycles based on them. The development of efficient and reliable methods for the formation of carbon–phosphorus bonds is of great importance in connection with the widespread use of organophosphorus compounds in chemistry, materials science and biology. In recent years, interest in functionalized organophosphorus compounds has been steadily increasing, as evidenced by a large number of publications in the field of their chemical and structural studies [86,87,88,89,90,91,92]. It should be noted that the skillful use of phosphorus pentachloride as a phosphorylating agent for derivatives of enamines, enamides, tertiary amines, acetyl ureas and other nucleophiles leads to the production of various *C*- and *N*-chlorophosphorylated compounds, which, under certain conditions, are transformed to form promising nitrogen- and phosphorus-containing heterocyclic systems of diverse structures. 

NMR spectroscopy studies of chlorophosphorylated enamines based on available tertiary amines and a wide range of other nitrogen-containing organic nucleophiles make a significant contribution to the development of the chemistry of unsaturated organophosphorus compounds. The use (involvement) of multipulse and multinuclear NMR spectroscopy, in particular ^31^P NMR, simplifies the tasks associated with establishing the structure of phosphorylation products and stereochemical behavior (e.g., for *E*,*Z*-isomeric compounds). Analysis of the ^31^P NMR chemical shifts of phosphorus–nitrogen-containing enamines and the corresponding heterocycles indicates that a variation in the coordination number of the phosphorus atom from 3 to 6 leads to a dramatic change in the screening of the ^31^P nucleus from about +200 to −300 ppm. 

^31^P NMR spectroscopy is the most convenient, powerful and simple express method for recognizing the coordination number of the phosphorus atom in organophosphorus compounds, as well as for determining (identifying) the structure of isomeric forms of organophosphorus products. A certain contribution to the determination of the structure of chlorophosphorylated compounds is made by ^35^Cl NQR spectroscopy. 

This review article can be considered as the first step towards a further deeper analysis of NMR spectroscopy and quantum-chemical data for a wide range of organic compounds, in particular, saturated and unsaturated compounds and heterocycles based on them. In this regard, it will be possible to apply the Karplus equation, which works mainly for saturated systems of the H–C(*sp*^3^)–C(*sp*^3^)–H type.

## Figures and Tables

**Table 1:** The ^1^H, ^13^C and ^31^P NMR chemical shifts (δ, ppm) and coupling constants (*J*, Hz) of phosphorylated enamines **1**–**13**.

No.	Structure	^1^H *^a^*	^13^C	^31^P *^b^*
**1**	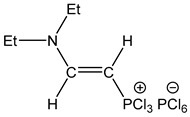	8.00 dd, 1H, N–CH= ^3^*J*_PH_ = 25.9, ^3^*J*_HH_ = 13.7 5.65 dd, 1H, P–CH= ^2^*J*_PH_ = 33.7, ^3^*J*_HH_ = 13.7	153.25 d, N–CH=, ^2^*J*_PC_ = 47.3 81.47 d, P–CH=, ^1^*J*_PC_ = 195.4	89.9 dd ${\mathrm{PCl}}_{3}^{+}$ ^2^*J*_PH_ = 33.7 ^3^*J*_PH_ = 25.9
**2**	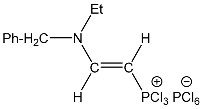	7.81 dd, 1H, N–CH= ^3^*J*_PH_ = 23.8, ^3^*J*_HH_ = 13.9 5.50 dd, 1H, P–CH= ^2^*J*_PH_ = 34.0, ^3^*J*_HH_ = 13.9	155.12 d, N–CH=, ^2^*J*_PC_ = 48.1 80.92 d, P–CH=, ^1^*J*_PC_ = 200.2	80.1 dd ${\mathrm{PCl}}_{3}^{+}$ ^2^*J*_PH_ = 34.0 ^3^*J*_PH_ = 23.8
**3**	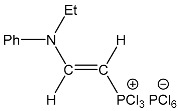	7.60 dd, 1H, N–CH= ^3^*J*_PH_ = 21.4, ^3^*J*_HH_ = 13.9 4.55 dd, 1H, P–CH= ^2^*J*_PH_ = 34.3, ^3^*J*_HH_ = 13.9	150.40 d, N–CH=, ^2^*J*_PC_ = 52.7 80.29 d, P–CH=, ^1^*J*_PC_ = 200.8	79.8 dd ${\mathrm{PCl}}_{3}^{+}$ ^2^*J*_PH_ = 34.3 ^3^*J*_PH_ = 21.4
**4**	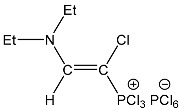	7.50 d, 1H, N–CH= ^3^*J*_PH_ = 14.1	158.11 d, N–CH=, ^2^*J*_PC_ = 37.5 77.26 d, P–CH=, ^1^*J*_PC_ = 187.1	80.7 d ${\mathrm{PCl}}_{3}^{+}$ ^3^*J*_PH_ = 14.1
**5**	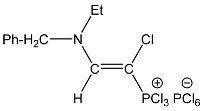	7.65 d, 1H, N–CH= ^3^*J*_PH_ = 13.9	157.20 d, N–CH=, ^2^*J*_PC_ = 35.4 77.82 d, P–CH=, ^1^*J*_PC_ = 179.5	82.0 d ${\mathrm{PCl}}_{3}^{+}$ ^3^*J*_PH_ = 13.9
**6**	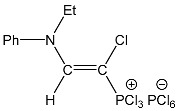	7.50 d, 1H, N–CH= ^3^*J*_PH_ = 13.7	159.21 d, N–CH=, ^2^*J*_PC_ = 30.9 75.64 d, P–CH=, ^1^*J*_PC_ = 159.6	72.0 d ${\mathrm{PCl}}_{3}^{+}$ ^3^*J*_PH_ = 13.7
**7**	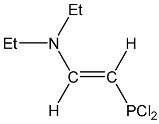	7.25 dd, 1H, N–CH= ^3^*J*_PH_ = 18.3, ^3^*J*_HH_ = 13.1 5.36 dd, 1H, P–CH= ^2^*J*_PH_ = 9.0, ^3^*J*_HH_ = 13.1 3.27 m 4H, CH_2 _, 1.29 m 3HCH_3_	156.80 d, N–CH=, ^2^*J*_PC_ = 31.0 85.11 d, P–CH=, ^1^*J*_PC_ = 201.2 50.67 s, CH_2_ 14.86 s, CH_3_	160.0 dd PCl_2_ ^2^*J*_PH_ = 9.0 ^3^*J*_PH_ = 18.3
**8**	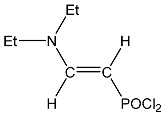	7.06 dd, 1H, N–CH= ^3^*J*_PH_ = 21.5, ^3^*J*_HH_ = 13.7 5.12 dd, 1H, P–CH= ^2^*J*_PH_ = 25.8, ^3^*J*_HH_ = 13.7 3.51 m 4H, CH_2, _ 1.27 m 6H, CH_3_	150.88 d, N–CH=, ^2^*J*_PC_ = 38.9 85.45 d, P–CH=, ^1^*J*_PC_ = 208.9 43.51 s, CH_2_ 13.84 s, CH_3_	35.0 dd POCl_2_ ^2^*J*_PH_ = 25.8 ^3^*J*_PH_ = 21.5
**9**	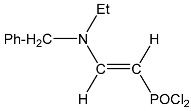	7.25 dd, 1H, N–CH= ^3^*J*_PH_ = 21.2, ^3^*J*_HH_ = 14.2 7.1–7.3 m, 5H, Ph 5.20 dd, 1H, P–CH= ^2^*J*_PH_ = 25.5, ^3^*J*_HH_ = 14.2 4.62 s, 2H, CH_2_-Ph 3.54 m, 2H, CH_2_ 1.28 m, 3H, CH_3_	149.27 d, N–CH=, ^2^*J*_PC_ = 37.1 86.23 d, P–CH=, ^1^*J*_PC_ = 208.9 125.20 m, Ph 51.60 s, CH_2_-Ph 49.70 s, CH_2_ 15.23 s, CH_3_	36.2 dd POCl_2_ ^2^*J*_PH_ = 25.5 ^3^*J*_PH_ = 21.2
**10**	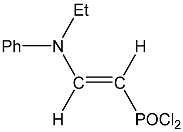	7.20 dd, 1H, N–CH= ^3^*J*_PH_ = 21.8, ^3^*J*_HH_ = 14.1 7.1–7.3 m, 5H, Ph 4.95 dd, 1H, P–CH= ^2^*J*_PH_ = 26.4, ^3^*J*_HH_ = 14.1 3.73 m, 2H, CH_2_ 1.25 m, 3H, CH_3_	150.10 d, N–CH=, ^2^*J*_PC_ = 32.8 85.32 d, P–CH=, ^1^*J*_PC_ = 201.4 120.40 m, Ph 48.34 s, CH_2_ 15.00 s, CH_3_	37.0 dd POCl_2_ ^2^*J*_PH_ = 26.4 ^3^*J*_PH_ = 21.8
**11**	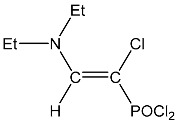	7.15 d, 1H, N–CH= ^3^*J*_PH_ = 11.6 3.45 m, 4H, CH_2 _ 1.18 m, 6H, CH_3_	145.19 d, N–CH=, ^2^*J*_PC_ = 39.4 ^1^*J*_CH=_165.7 88.64 d, P–CH=, ^1^*J*_PC_ = 212.5 46.91 s, CH_2_ 14.84 s, CH_3_	36.1 d POCl_2_ ^3^*J*_PH_ = 11.6
**12**	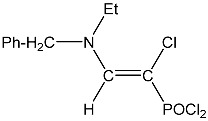	7.34 d, 1H, N–CH= ^3^*J*_PH_ = 12.1 7.2–7.4 m, 5H, Ph 5.37 s, 2H, CH_2_-Ph 3.53 m, 2H, CH_2 _ 1.23 m, 3H, CH_3_	148.95 d, N–CH=, ^2^*J*_PC_ = 39.8 86.02 d, P–CH=, ^1^*J*_PC_ = 210.3 124.24 m, Ph 54.26 s, CH_2_ -Ph 49.15 s, CH_2_ 14.65 s, CH_3_	35.9 d POCl_2_ ^3^*J*_PH_ = 12.1
**13**	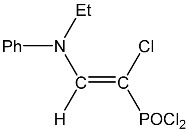	7.51 d, 1H, N–CH= ^3^*J*_PH_ = 11.1 7.1–7.3 m, 5H, Ph 3.55 m, 2H, CH_2 _ 1.20 m, 3H, CH_3_	151.44 d, N–CH=, ^2^*J*_PC_ = 33.5 84.57 d, P–CH=, ^1^*J*_PC_ = 200.4 127.40 m, Ph 51.34 s, CH_2_ 15.07 s, CH_3_	34.8 d POCl_2_ ^3^*J*_PH_ = 11.1

*^a^* It is impossible to identify the signals of protons of both ethyl and phenyl groups in compounds **1**–**6** (hexachlorophosphorates), since they and the tertiary amine hydrochlorides present in the crystalline reaction product have similar ethyl and phenyl substituents (δ^1^H: 1.20–1.28, 3.2–3.5 and 7.2–7.5 ppm, respectively, CH_3_, CH_2_, Ph). *^b^* The phosphorus signal of ${\mathrm{PCl}}_{6}^{-}$ anion in the ^31^P NMR spectra of organyltrichlorophosphonium hexachlorophosphorates **1**–**6** appears in the region −(297–298) ppm. For (**13a**) Ph_2_N—CH=C(Cl)-POCl_2_, the value of δ ^31^P is 34.6 ppm with constant ^3^*J*_PH_ = 11.6 Hz.

**Table 2:** The ^1^H, ^13^C and ^31^P NMR chemical shifts (δ, ppm) and coupling constants (*J*, Hz) of phosphorylated enamines **14**–**18**.

No.	Structure	^1^H	^13^C	^31^P
**14**	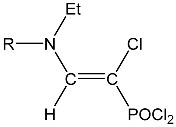	7.47 d, 1H, N–CH= ^3^*J*_PH_ = 11.8 7.3 m, 5H, Ph 3.02 s, 3H, CH_3_	148.40 d, N–CH=, ^2^*J*_PC_ = 30.5 123.4 m Ph 81.37 d, P–CH=, ^1^*J*_PC_ = 201.0 18.07 CH_3_	34.2 d POCl_2_ ^3^*J*_PH_ = 11.8
**15**	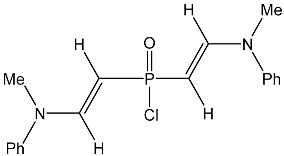	7.63 dd, 1H, N–CH= ^3^*J*_PH_ = 16.4, ^3^*J*_HH_ = 12.0 7.18 m, 5H, Ph 6.68 dd, 1H, P–CH= ^3^*J*_HH_ = 12.0, ^2^*J*_PH_ = 5.1 3.11 s, 3H, CH_3_	150.20 d, N–CH=, ^2^*J*_PC_ = 36.9 123.40 m Ph 88.11 d, P–CH=, ^1^*J*_PC_ = 140.9 15.12 CH_3_	35.6 tt POCl ^3^*J*_PH_ = 16.4 ^2^*J*_PH_ = 5.1
**16**	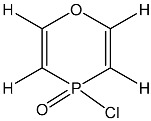	7.59 dd, 1H, O–CH= ^3^*J*_PH_ = 31.4, ^3^*J*_HH_ = 6.4 5.68 dd, 1H, P–CH= ^3^*J*_HH_ = 6.4, ^2^*J*_PH_ = 1.5	149.27 d, O–CH = 120.23 d, P–CH= ^1^*J*_PC_ = 120.9	10.7 tt POCl ^3^*J*_PH_ = 31.4 ^2^*J*_PH_ = 1.5
**17**	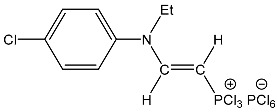	7.28 dd, 1H, N–CH= ^3^*J*_PH_ = 22.9, ^3^*J*_HH_ = 13.8 7.30 m, 4H, Ph 4.90 dd, 1H, P–CH= ^2^*J*_PH_ = 33.6, ^3^*J*_HH_ = 13.8 3.53 m, 2H, CH_2_ 1.21 m, 3H, CH_3_	156.10 d, N–CH=, ^2^*J*_PC_ = 32.8 129.24 m Ph 85.32 d, P–CH=, ^1^*J*_PC_ = 201.4 48.34 CH_2_ 15.00 CH_3_	82.7 dd ${\mathrm{PCl}}_{3}^{+}$ ^2^*J*_PH_ = 33.6 ^3^*J*_PH_ = 22.9 −297.9 br s ${\mathrm{PCl}}_{6}^{-}$
**18**	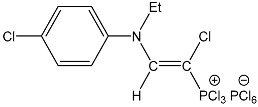	7.45 d, 1H, N–CH= ^3^*J*_PH_ = 14.5 7.24 m, 4H, Ph 3.41 m, 2H, CH_2 _ 1.16 m, 3H, CH_3_	145.19 d, N–CH=, ^2^*J*_PC_ = 39.6 86.64 d, P–CH=, ^1^*J*_PC_ = 200.5 46.91 CH_2_ 14.84 CH_3_	74.0 d PCl_3_ ^3^*J*_PH_ = 14.5 −298.0 br s ${\mathrm{PCl}}_{6}^{-}$

**Table 3:** The ^1^H, ^13^C and ^31^P NMR chemical shifts (δ, ppm) and coupling constants (*J*, Hz) of phosphorylated compounds **19**–**24**.

No.	Structure	^1^H	^13^C	^31^P
**19**	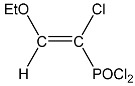	7.34 d, 1H, CH= ^3^*J*_PH_ = 18.3 4.50 m, 2H, CH_2_ 1.52 m, 3H, CH_3_	135.30 s, CH= 125.76 d, CCl ^1^*J*_PC _= 92.2 42.54 s, OCH_2_ 15.32 s, CH_3_	80.1 d ^3^*J*_PH _= 18.3
**20**	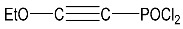	4.42 m, 2H, CH_2_ 1.50 m, 3H, CH_3_	102.30 CO 94.30 CP 44.50 OCH_2_ 13.40 CH_3_	6.0 s
**21**	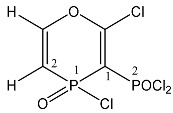	8.15 ddd, 1H, O–CH= ^3^*J*_P1H _= 57.2, ^5^*J*_P2H_ = 19.1 ^3^*J*_HH_ = 11.0 7.59 ddd, 1H, P–CH= ^4^*J*_P2H _= 40.4, ^2^*J*_P1H_ = 27.0 ^3^*J*_HH_ = 11.0 4.30 m, 2H, CH_2_ 1.55 m, 3H, CH_3_	138.5 d, C-2 ^1^*J*_P1C2 _= 101.2 106.4 dd, C-1 ^1^*J*_P1C1 _= 157.7 ^1^*J*_P2C1 _= 67.5	28.3 ddd, P1 ^3^*J*_P1H _= 57.2, ^2^*J*_PP _= 56.5, ^2^*J*_P1H_ = 27.0 21.4 ddd P2 ^2^*J*_PP _= 56.5, ^4^*J*_P2H_ = 40.4 ^5^*J*_P2H _= 19.1
**22**	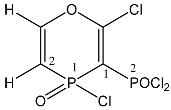	8.20 ddd, 1H, O–CH= ^3^*J*_P1H _= 62.0, ^5^*J*_P2H_ = 16.8 ^3^*J*_HH_ = 11.0 7.50 d, 1H, P–CH= ^4^*J*_P2H _= 36.6, ^2^*J*_P1H_ = 27.5 ^3^*J*_HH_ = 11.0	139.1 d, C-2 ^1^*J*_P1C2_ = 100.0 108.7 dd, C-1 ^1^*J*_P1C1 _= 150.0 ^1^*J*_P2C1 _= 68.0	25.6 ddd P1 ^3^*J*_P1H_ = 62.0 ^2^*J*_PP _= 48.8, ^2^*J*_P1H_ = 27.5 21.9 ddd P2 ^2^*J*_PP _= 48.8, ^4^*J*_P2H_ = 36.6 ^5^*J*_P2H_ = 16.8
**23**	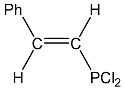	7.58 dd, 1H, P–CH= ^3^*J*_HH_ = 15.3, ^2^*J*_PH _= 9.2 7.50 m, 5H, Ph 7.30 dd, 1H, Ph–CH= ^3^*J*_PH _= 19.1, ^3^*J*_HH_ = 15.3	142.2 d, C-P ^1^*J*_PC_ = 98.0 112.4 d, C-Ph ^2^*J*_PC _= 35.0	162.8 dd PCl_2_ ^3^*J*_PH _= 19.1 ^2^*J*_PH _= 9.2
**24**	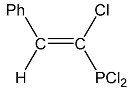	7.50 m, 5H, Ph 7.40 d, 1H, Ph–CH= ^3^*J*_PH _= 22.1	142.2 d, C-P ^1^*J*_PC _= 99.0 118.3 d, C-Ph ^2^*J*_PC_ = 33.0	157.8 d PCl_2_ ^3^*J*_PH_ = 22.1

**Table 4:** The ^1^H and ^31^P NMR chemical shifts (δ, ppm) and coupling constants (*J*, Hz) of phosphorylated enamides **25**–**33** (CH_3_NO_2_) and **34**–**42** (CDCl_3_).

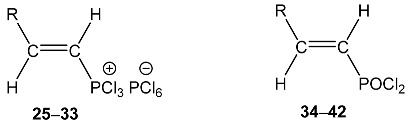					
R	No.	^31^P ^a^, ${\mathrm{PCl}}_{3}^{+}\;$	No.	^1^H	^31^P, POCl_2_
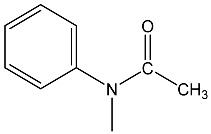	**25**	94.1 dd ^2^*J*_PH _= 40.2 ^3^*J*_PH _= 25.9	**34**	8.50 dd, 1H, N–CH= ^3^*J*_PH_ = 21.8, ^3^*J*_HH_ = 15.0 7.32 m, 5H, Ph 4.75 dd, 1H, P–CH= ^2^*J*_PH_ = 27.8, ^3^*J*_HH_ = 15.0 1.91 3H, CH_3_	34.7 dd ^2^*J*_PH _= 27.8 ^3^*J*_PH _= 21.8
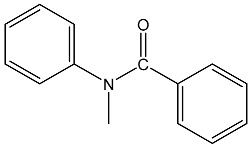	**26**	93.7 dd ^2^*J*_PH _= 40.0 ^3^*J*_PH _= 26.0	**35**	8.54 dd, 1H, N–CH= ^3^*J*_PH_ = 21.9, ^3^*J*_HH_ = 15.0 7.35 m, 10H, Ph 5.12 dd, 1H, P–CH= ^2^*J*_PH_ = 27.8, ^3^*J*_HH_ = 15.0	33.7 dd ^2^*J*_PH _= 27.8 ^3^*J*_PH _= 21.9
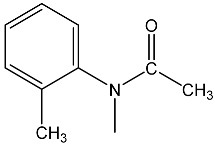	**27**	94.0 dd ^2^*J*_PH _= 39.6 ^3^*J*_PH _= 26.6	**36**	7.81 dd, 1H, N–CH= ^3^*J*_PH_ = 21.9, ^3^*J*_HH_ = 15.2 7.20 m, 4H, Ph 5.00 dd, 1H, P–CH= ^2^*J*_PH_ = 27.8, ^3^*J*_HH_ = 15.2 2.15, 3H, CH_3_ 1.98 3H, CH_3_	34.7 dd ^2^*J*_PH _= 27.8 ^3^*J*_PH _= 21.9
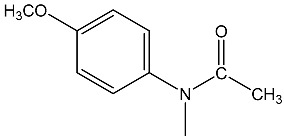	**28**	94.0 dd ^2^*J*_PH _= 40.0 ^3^*J*_PH _= 26.1	**37**	8.32 dd, 1H, N–CH= ^3^*J*_PH_ = 21.5, ^3^*J*_HH_ = 15.1 7.33 m, 4H, Ph 4.88 dd, 1H, P–CH= ^2^*J*_PH_ = 28.5, ^3^*J*_HH_ = 15.1 4.15 3H, OMe; 1.96 3H, CH_3_	34.1 dd ^2^*J*_PH _= 28.5 ^3^*J*_PH _= 21.5
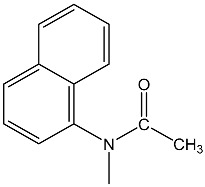	**29**	93.5 dd ^2^*J*_PH _= 40.0 ^3^*J*_PH _= 26.0	**38**	8.77 dd, 1H, N–CH= ^3^*J*_PH_ = 21.6, ^3^*J*_HH_ = 14.6 8.1–7.4 m, 7H, Napht 4.72 dd, 1H, P–CH= ^2^*J*_PH_ = 27.9, ^3^*J*_HH_ = 14.6 1.88 3H, CH_3_	33.7 dd ^2^*J*_PH _= 27.9 ^3^*J*_PH _= 21.6
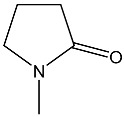	**30**	92.1 dd ^2^*J*_PH _= 39.7 ^3^*J*_PH _= 26.8	**39**	7.84 dd, 1H, N–CH= ^3^*J*_PH_ = 21.7, ^3^*J*_HH_ = 15.1 5.30 dd, 1H, P–CH= ^2^*J*_PH_ = 26.9, ^3^*J*_HH_ = 15.1 3.53 m, 2H, NCH_2_ 2.49 m, 2H, CH_2_CO 2.1 m, 2H, CH_2_	33.9 dd ^2^*J*_PH _= 26.9 ^3^*J*_PH _= 21.7
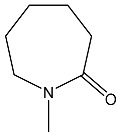	**31**	92.5 dd ^2^*J*_PH _= 40.4 ^3^*J*_PH _= 26.3	**40**	8.12 dd, 1H, N–CH= ^3^*J*_PH_ = 24.0, ^3^*J*_HH_ = 15.2 5.41 dd, 1H, P–CH= ^2^*J*_PH_ = 26.5, ^3^*J*_HH_ = 15.2 3.55 m, 2H, NCH_2_ 2.58 m, 2H, CH_2_CO 1.6 m, 6H, CH_2_	37.3 dd ^2^*J*_PH _= 26.5 ^3^*J*_PH _= 24.0
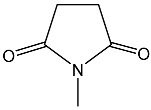	**32**	98.9 dd ^2^*J*_PH_ = 40.0 ^3^*J*_PH_ = 29.4	**41**	7.53 dd, 1H, N–CH= ^3^*J*_PH_ = 26.2, ^3^*J*_HH_ = 15.6 7.31 dd, 1H, P–CH= ^2^*J*_PH_ = 30.5, ^3^*J*_HH_ = 15.6 2.84 m, 4H, CH_2_CO	33.1 dd ^2^*J*_PH_ = 30.5 ^3^*J*_PH _= 26.2
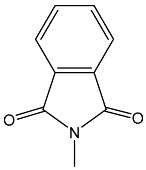	**33**	94.0 dd ^2^*J*_PH _= 39.6 ^3^*J*_PH _= 26.6	**42**	7.64 dd, 1H, N–CH= ^3^*J*_PH_ = 25.4, ^3^*J*_HH_ = 15.6 7.85 m, 4H, Ph 7.12 dd, 1H, P–CH= ^2^*J*_PH_ = 30.1, ^3^*J*_HH_ = 15.6	34.4 dd ^2^*J*_PH _= 30.1 ^3^*J*_PH _= 25.4

^a^ The phosphorus signal of ${\mathrm{PCl}}_{6}^{-}$ anion in the ^31^P NMR spectra of hexachlorophosphorates **25**–**33** appears in the region −(296–298) ppm; the ^3^*J*_HH_ value varies within 15–16 Hz.

**Table 5:** The ^1^H, ^13^C and ^31^P NMR chemical shifts (δ, ppm) and coupling constants (*J*, Hz) of phosphonic dichlorides **43** and **45** (CDCl_3_) and hexachlorophosphorate (**44**) (CH_3_NO_2_).

No.	R	^1^H	^13^C	^31^P
**43**	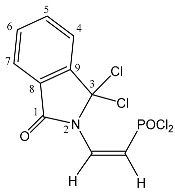	7.80 m, 4H, H-(4–7) 7.10 dd, 1H, N–CH= ^3^*J*_PH_ = 57.1, ^3^*J*_HH_ = 10.5 6.28 dd, 1H, P–CH= ^2^*J*_PH _= 26.2, ^3^*J*_HH_ = 10.5	157.70 C-1 140.98 C-9 131.14 C-4 128.20 d, N–CH= ^2^*J*_PC_ = 8.5 127.53 C-7 121.05 C-8 119.64 C-6 118.57 C-5 114.55 d, P–CH= ^1^*J*_PC_ = 151.4 86.77 C-3	23.3 dd ^2^*J*_PH _= 26.2 ^3^*J*_PH _= 57.1
**44**	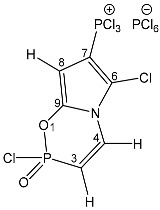	7.78 dd, 1H, N–CH= ^3^*J*_PH_ = 40.8, ^3^*J*_HH_ = 10.0 6.44 dd, 1H, P–CH= ^2^*J*_PH _= 20.0, ^3^*J*_HH_ = 10.0 6.22 d, 1H, O–CH= ^3^*J*_HH _= 6.0	142.60 dd, C-9 ^2^*J*_PC_ = 23.3 d, ^3^*J*_PC_ = 10.0 133.40 d, C-4, ^2^*J*_PC_ = 6.0 117.84 d, C-6, ^2^*J*_PC_ = 16.2 113.75 d, C-7, ^1^*J*_PC_ = 185.3 101.50 dd, C-8 ^2^*J*_PC_ = 16.6 d, ^3^*J*_PC_ = 5.5	83.5 dd ${\mathrm{PCl}}_{3}^{+}$ ^3^*J*_PH _= 6.0 ^5^*J*_PP _= 2.8 11.1 ddd POCl ^3^*J*_PH_ = 40.8 ^2^*J*_PH_ = 20.0 ^4^*J*_PH_ = 2.8 −297.7 br s ${\mathrm{PCl}}_{6}^{-}$
**45**	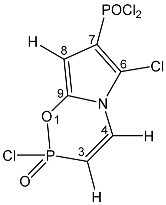	7.67 dd, 1H, N–CH= ^3^*J*_PH_ = 40.5, ^3^*J*_HH_ = 10.5 6.17 dd, 1H, P–CH= ^2^*J*_PH _= 18.6, ^3^*J*_HH_ = 10.5 6.23 d, 1H, O–CH= ^3^*J*_HH _= 6.1	139.82 dd, C-9 ^2^*J*_PC_ = 25.6 d, ^3^*J*_PC_ = 11.0 135.29 d, C-4 ^2^*J*_PC_ = 6.1 115.95 d, C-6, ^2^*J*_PC_ = 18.3 114.80 d, C-7, ^1^*J*_PC_ = 194.7 96.50 dd, C-8 ^2^*J*_PC_ = 14.6 d, ^3^*J*_PC_ = 4.5	18.6 dd POCl_2_ ^3^*J*_PH_ = 6.1 ^5^*J*_PP_ = 2.8 10.4 ddd POCl ^3^*J*_PH _= 40.5 ^2^*J*_PH _= 18.6 ^4^*J*_PH _= 2.8

**Table 6:** The ^1^H and ^31^P NMR chemical shifts (δ, ppm) and coupling constants (*J*, Hz) of phosphorylated acetamides **53**–**57** (CH_3_NO_2_) and **58**–**62** (CDCl_3_).

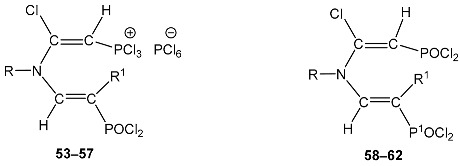								
No.	R^1^	R	^31^P, POCl_2_	^31^P ^a^, ${\mathrm{PCl}}_{3}^{+}\;$	No.	^1^H	^31^P, P^1^OCl_2_	^31^P, POCl_2_
**53**	H	Pr	31.2 dd ^2^*J*_PH_ = 23.4 ^3^*J*_PH_ = 23.4	82.7 d ^2^*J*_PH_ = 32.2	**58**	7.50 dd, 1H, N–CH= ^3^*J*_PH_ = 23.0, ^3^*J*_HH_ = 15.0 5.75 dd, 1H, P–CH= ^2^*J*_PH_ = 17.0 5.48 dd, 1H, P^1^–CH= ^2^*J*_PH_ = 23.0, ^3^*J*_HH_ = 15.0 3.64 t, NCH_2_, ^3^*J*_HH_ = 6.7 1.49 m, 2H, CH_2_ 0.98 t, 3H, CH_3_, ^3^*J*_HH_ = 6.9	33.7 dd ^2^*J*_PH_ = 23.0 ^3^*J*_PH_ = 23.0	25.4 d ^2^*J*_PH_ = 17.0
**54**	H	Bu	30.7 dd ^2^*J*_PH_ = 22.4 ^3^*J*_PH_ = 22.4	81.5 d ^2^*J*_PH_ = 30.3	**5** **9**	7.73 dd, 1H, N–CH= ^3^*J*_PH_ = 21.8, ^3^*J*_HH_ = 14.8 5.78 d, 1H, P–CH= ^2^*J*_PH_ = 17.1 5.40 dd, 1H, P^1^–CH= ^2^*J*_PH_ = 23.9, ^3^*J*_HH_ = 14.8 3.62 t, NCH_2_, ^3^*J*_HH_ = 6.8 1.44 m, 4H, (CH_2_)_2_ 0.92 t, 3H, CH_3_, ^3^*J*_HH_ = 6.7	32.5 dd ^2^*J*_PH_ = 23.9 ^3^*J*_PH_ = 21.8	24.1 d ^2^*J*_PH_ = 17.1
**55**	H	PhCH_2_	30.2 dd ^2^*J*_PH_ = 24.2 ^3^*J*_PH_ = 23.4	81.0 d ^2^*J*_PH_ = 30.6	**6** **0**	7.54 dd, 1H, N–CH= ^3^*J*_PH_ = 26.0, ^3^*J*_HH_ = 15.2 7.28 m, 5H, Ph 5.69 d, 1H, P–CH= ^2^*J*_PH_ = 16.2 5.48 dd, 1H, P^1^–CH= ^2^*J*_PH_ = 26.0, ^3^*J*_HH_ = 15.2 4.68 s, 2H, NCH_2_	33.0 dd ^2^*J*_PH_ = 26.0 ^3^*J*_PH_ = 26.0	24.7 d ^2^*J*_PH_ = 16.2
**56**	H	Ph	30.0 dd ^2^*J*_PH_ = 22.5 ^3^*J*_PH_ = 22.5	83.1 d ^2^*J*_PH_ = 31.8	**61**	7.70 dd, 1H, N–CH= ^3^*J*_PH_ = 20.3, ^3^*J*_HH_ = 14.8 7.22 m, 5H, Ph 5.70 d, 1H, P–CH= ^2^*J*_PH_ = 18.0 5.47 dd, 1H, P^1^–CH= ^2^*J*_PH_ = 25.0, ^3^*J*_HH_ = 14.8	33.4 dd ^2^*J*_PH_ = 25.0 ^3^*J*_PH_ = 20.3	26.0 d ^2^*J*_PH_ = 18.0
**57**	Et	PhCH_2_	31.3 dd ^3^*J*_PH_ = 28.6 CH_2_ (Et) ^3^*J*_PH_ = 19.7	75.5 d ^2^*J*_PH_ = 30.5	**62**	7.49 d, 1H, N–CH= ^3^*J*_PH_ = 21.6 7.20 m, 5H, Ph 5.62 d, 1H, P–CH= ^2^*J*_PH_ = 18.7 4.23 s, 2H, NCH_2_ 3.28 d, 2H, CH_2_^3^*J*_PH_ = 28.6 1.70 3H, CH_3_	38.7 dd ^3^*J*_PH_ = 28.6 CH_2_ (Et) ^3^*J*_PH_ = 21.6	24.5 d ^2^*J*_PH_ = 18.7

^a^ The phosphorus signal of ${\mathrm{PCl}}_{6}^{-}\;$ anion in the ^31^P NMR spectra of hexachlorophosphorates **53–57** appears in the region −(297–298) ppm.

**Table 7:** The ^1^H and ^31^P NMR chemical shifts (δ, ppm) and coupling constants (*J*, Hz) of hexachlorophosphorates **63–67** (CH_3_NO_2_) and 1,4-azaphosphorines **68–72** (CDCl_3_).

No.	**R**	**^1^H**	**^31^P, POCl_2_**	^31^P ^a^, ${\mathrm{PCl}}_{2}^{+}$/POCl	**^2^** ***J*** **_PP_**
**63**	Pr	9.65 dd, 1H, N–CH= ^3^*J*_PH_ = 45.1, ^3^*J*_PH_ = 22.9 8.25 dd, 1H, P–CH=, ^4^*J*_PH_ = 10.4 3.64 t, NCH_2_, ^3^*J*_HH_ = 6.6 1.49 m, 2H, CH_2_ 0.98 t, 3H, CH_3_, ^3^*J*_HH_ = 6.8	19.8 ddd ^2^*J*_PP _= 25.9 ^3^*J*_PH _= 22.9 ^4^*J*_PH _= 10.4	46.8 dd ^3^*J*_PH _= 45.1 ^2^*J*_PP _= 25.9	25.9
** 64 **	Bu	9.90 dd, 1H, N–CH= ^3^*J*_PH_ = 45.5, ^3^*J*_PH_ = 22.3 8.28 d, 1H, P–CH=, ^4^*J*_PH_ = 10.1 4.18 t, NCH_2_, ^3^*J*_HH_ = 6.6 1.44 m, 1.72 m, 4H, (CH_2_)_2_ 0.90 t, 3H, CH_3_, ^3^*J*_HH_ = 6.7	20.3 ddd ^2^*J*_PP _= 25.5 ^3^*J*_PH _= 22.3 ^4^*J*_PH _= 10.1	46.2 dd ^3^*J*_PH _= 45.5 ^2^*J*_PP _= 25.5	25.5
** 65 **	PhCH_2_	9.85 dd, 1H, N–CH= ^3^*J*_PH_ = 45.0, ^3^*J*_PH_ = 22.3 8.02 d, 1H, P–CH=, ^4^*J*_PH_ = 9.9 7.27 m, 5H, Ph 4.47 s, 2H, NCH_2_	19.5 ddd ^2^*J*_PP _= 25.6 ^3^*J*_PH _= 22.3 ^4^*J*_PH _= 9.9	46.4 dd ^3^*J*_PH _= 45.0 ^2^*J*_PP _= 25.6	25.6
** 66 **	Ph	8.77 dd, 1H, N–CH= ^3^*J*_PH_ = 44.6, ^3^*J*_PH_ = 24.3 7.21 d, 1H, P–CH=, ^4^*J*_PH_ = 9.6 7.34 m, 5H, Ph	20.0 ddd ^2^*J*_PP _= 25.0 ^3^*J*_PH _= 24.8 ^4^*J*_PH _= 9.6	48.1 dd ^3^*J*_PH _= 44.6 ^2^*J*_PP _= 25.0	25.0
** 67 **	s-Bu	8.85 dd, 1H, N–CH= ^3^*J*_PH_ = 45.0, ^3^*J*_PH_ = 26.0 7.40 d, 1H, P–CH=, ^4^*J*_PH_ = 10.5 3.34 m, 1H, NCH 1.5 m, 2H, CH_2_ 1.15 m, 6H, CH_3_	20.6 ddd ^2^*J*_PP _= 26.0 ^3^*J*_PH _= 26.0 ^4^*J*_PH _= 10.5	45.5 dd ^3^*J*_PH _= 45.0 ^2^*J*_PP _= 26.0	26.0
**68**	Pr	8.12 dd, 1H, N–CH= ^3^*J*_PH_ = 33.0, ^3^*J*_PH_ = 23.9 6.68 d, 1H, P–CH=, ^4^*J*_PH_ = 10.9 3.40 t, NCH_2_, ^3^*J*_HH_ = 6.4 1.53 m, 2H, CH_2_ 1.04 t, 3H, CH_3_, ^3^*J*_HH_ = 6.7	22.6 ddd ^2^*J*_PP _= 25.9 ^3^*J*_PH _= 23.9 ^4^*J*_PH _= 10.9	10.9 dd ^3^*J*_PH _= 33.0 ^2^*J*_PP _= 25.9	25.9
**69**	Bu	8.07 dd, 1H, N–CH= ^3^*J*_PH_ = 32.9, ^3^*J*_PH_ = 23.5 6.22 d, 1H, P–CH=, ^4^*J*_PH_ = 10.3 4.05 t, NCH_2_, ^3^*J*_HH_ = 6.5 1.40 m, 1.75 m, 4H, (CH_2_)_2_ 0.92 t, 3H, CH_3_, ^3^*J*_HH_ = 6.4	22.1 ddd ^2^*J*_PP _= 24.3 ^3^*J*_PH _= 23.5 ^4^*J*_PH _= 10.3	10.2 dd ^3^*J*_PH _= 32.9 ^2^*J*_PP _= 24.3	24.3
**70**	PhCH_2_	8.07 dd, 1H, N–CH= ^3^*J*_PH_ = 32.4, ^3^*J*_PH_ = 22.9 7.48 m, 5H, Ph 6.24 d, 1H, P–CH=, ^4^*J*_PH_ = 10.2 4.24 s, 2H, NCH_2_	25.5 ddd ^2^*J*_PP _= 24.4 ^3^*J*_PH _= 22.9 ^4^*J*_PH _= 10.2	13.5 dd ^2^*J*_PP _= 24.4 ^3^*J*_PH _= 32.4	24.4
**71**	Ph	7.95 dd, 1H, N–CH= ^3^*J*_PH_ = 32.6, ^3^*J*_PH_ = 22.8 7.44 m, 5H, Ph 6.18 d, 1H, P–CH=, ^4^*J*_PH_ = 9.9	25.8 ddd ^2^*J*_PP _= 24.1 ^3^*J*_PH _= 22.8 ^4^*J*_PH _= 9.9	13.9 dd ^2^*J*_PP _= 24.1 ^3^*J*_PH _= 32.6	24.1
**72**	s-Bu	7.90 dd, 1H, N–CH= ^3^*J*_PH_ = 32.2, ^3^*J*_PH_ = 23.4 6.47 d, 1H, P–CH=, ^4^*J*_PH_ = 10.8 3.44 m, 1H, NCH 1.5 m, 2H, CH_2_ 1.13 m, 6H, CH_3_	24.0 ddd ^2^*J*_PP _= 24.6 ^3^*J*_PH _= 23.4 ^4^*J*_PH _= 10.8	13.1 dd ^2^*J*_PP _= 24.6 ^3^*J*_PH _= 32.2	24.6

^a^ The ${\mathrm{PCl}}_{6}^{-}$ anion in organyltrichlorophosphonium hexachlorophosphorates **63–67** has a broad signal in the ^31^P NMR spectra in the region −(296–298) ppm.

**Table 8:** The ^1^H and ^31^P NMR chemical shifts (δ, ppm) and coupling constants (*J*, Hz) of hexachlorophosphorates **73** and **75** (CH_3_NO_2_) and 1,4-azaphosphorines **74** and **76** (CDCl_3_).

No.	^1^H/^13^C	^31^P, POCl_2_	^31^P, ${\mathrm{PCl}}_{2}^{+}/\mathrm{POCl}$
**73**	7.34 d, 1H, CH=, ^4^*J*_PH_ = 9.3 4.32 s, CH_3_ 140.8 d, CCl=, ^2^*J*_PC_ = 14.0 108.47 dd, P-C-P, ^1^*J*_PC_ = 152.6 (POCl_2_) ^1^*J*_PC_ = 101.3 (POCl) 106.20 dd, CH=, ^1^*J*_PC_ = 124.4, ^3^*J*_PC_ = 8.5 42.15 s, CH_3_	21.8 dd ^2^*J*_PP_ = 21.5 ^4^*J*_PH_ = 9.3	49.2 d ^2^*J*_PP_ = 21.5
** 74 **	6.24 d, 1H, CH=, ^4^*J*_PH_ = 8.5 3.80 s, CH_3_ 148.8 d, CCl=, ^2^*J*_PC_ = 12.0 110.24 dd, P-C-P, ^1^*J*_PC_ = 149.6 (POCl_2_) ^1^*J*_PC_ = 102.5 (POCl) 104.34 ddd, CH=, ^1^*J*_CH_ = 180.6, ^1^*J*_PC_ = 118.2, ^3^*J*_PC_ = 9.2 43.67 s, CH_3_	17.2 dd ^2^*J*_PP_ = 21.2 ^4^*J*_PH_ = 8.5	9.0 d ^2^*J*_PP_ = 21.2
** 75 **	11.37 br s, 1H, NH 5.98 d, 2H, CH=, ^2^*J*_PH_ = 2.2 145.20 d, CCl=, ^2^*J*_PC_ = 16.1 82.31 dd, CH=, ^1^*J*_CH_ = 187.0, ^1^*J*_PC_ = 110.8	-	49.9 t ^2^*J*_PH_ = 2.2
** 76 **	10.60 br s, 1H, NH 5.72 d, 2H, CH=, ^2^*J*_PH_ = 1.1 143.70 d, CCl=, ^2^*J*_PC_ = 14.2 80.44 dd, CH=, ^1^*J*_CH_ = 180.0, ^1^*J*_PC_ = 103.6	-	25.8 t ^2^*J*_PH _= 1.1

**Table 9:** The ^1^H, ^13^C and ^31^P NMR chemical shifts (δ, ppm) and coupling constants (*J,* Hz) of compounds **88** and **89** (CDCl_3_).

No.	Structure	^1^H	^13^C	^31^P
**88**	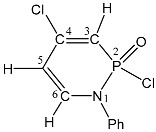	7.07–7.20 m, 5H, Ph 6.44 dd, 1H, H-6 ^3^*J*_PH_ = 23.9, ^3^*J*_HH_ = 8.1 5.85 dd, 1H, H-3, ^3^*J*_PH_ = 11.4, ^4^*J*_HH_ = 2.2 5.36 ddd, 1H, H-5, ^3^*J*_HH_ = 8.1, ^4^*J*_HH_ = 2.2, ^4^*J*_PH_ = 2.2	147.5, 129.8, 120.0, 127.2 Ph 139.5 d, C-6, ^2^*J*_PC_ = 2.0 138.3 s, C-Cl, 107, d, C-3, ^1^*J*_PC_ = 149.6 105.6 d, C-5, ^3^*J*_PC_ = 11.6	21.6 dd ^3^*J*_PH _= 23.9 ^2^*J*_PH_ = 11.4
** 89 **	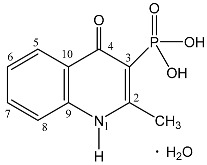	13.39 s, 1H, NH 8.22 d, 1H, H-5, ^3^*J*_HH_ = 8.2 7.89 dd, 1H, H-7, ^3^*J*_HH_ = 8.2, ^3^*J*_HH_ = 7.6 7.78 d, 1H, H-8, ^3^*J*_HH_ = 8.2 7.59 dd, 1H, H-6, ^3^*J*_HH_ = 8.2, ^3^*J*_HH_ = 7.6 3.8 br s, 2H, OH 2.83 s, 3H, CH_3_	176.03 d, C-4, ^2^*J*_PC_ = 7.7 157.24 d, C-2, ^2^*J*_PC_ = 13.8 138.66 s, C-9, 133.91 s, C-7, 126.05 s, C-5, 124.87 s, C-6, 121.17 d, C-10, ^3^*J*_PC_ = 11.1 118.86 s, C-8, 109.39 d, C-3, ^1^*J*_PC_ = 161.0 20.42 s, CH_3_	11.6 s

**Table 10:** The ^1^H, ^13^C and ^31^P NMR chemical shifts (δ, ppm) and coupling constants (*J,* Hz) of compounds **90**–**94**.

No.	R^1^	R	^1^H	^13^C	^31^P
**90**	H	Me	7.80 dd, 1H, H-3 ^3^*J*_HH_ = 15.2, ^4^*J*_PH_ = 2.9 7.21 d, 1H, H-4, ^3^*J*_HH_ = 15.2 5.30 d, 1H, H-1, ^2^*J*_PH_ = 56.4 2.10 s, 3H, CH_3_	143.50 d, C-2, ^2^*J*_PC_ = 27.8 133.85 s, C-4, 128.67, d, C-3, ^3^*J*_PC_ = 9.8 118.20 d, C-1, ^1^*J*_PC_ = 116.3 21.28 s CH_3_	84.3 dd ^2^*J*_PH_ = 56.4 ^4^*J*_PH_ = 2.9
** 91 **	Me	Me	7.73 d, 1H, H-3, ^4^*J*_PH_ = 2.7 5.61 d, 1H, H-1, ^2^*J*_PH_ = 56.2 2.08 s, 3H, CH_3_ 1.98 s, 3H, CH_3_	142.05 d, C-2, ^2^*J*_PC_ = 26.7 139.75 s, C-4, 124.51, d, C-3, ^3^*J*_PC_ = 7.2 116.50 d, C-1, ^1^*J*_PC_ = 114.9 29.27 s CH_3_ 21.66 s CH_3_	85.7 dd ^2^*J*_PH_ = 56.2 ^4^*J*_PH_ = 2.7
**92**	H	Ph	7.75 dd, 1H, H-3 ^3^*J*_HH_ = 14.8, ^4^*J*_PH_ = 3.1 7.55–7.40 m, 5H, Ph 7.15 d, 1H, H-4, ^3^*J*_HH_ = 14.8 5.87 d, 1H, H-1, ^2^*J*_PH_ = 41.0	144.65 d, C-2, ^2^*J*_PC_ = 26.0 140.90 s, i-Ph 136.86 s, C-4 128.98 s, m-Ph, p-Ph 127.69 s, o-Ph 127.48 d, C-3, ^3^*J*_PC_ = 8.9 117.27 d, C-1, ^1^*J*_PC_ = 115.5	89.1 dd ^2^*J*_PH_ = 41.0 ^4^*J*_PH_ = 3.1
** 93 **	H	p-MeO-Ph	7.80 dd, 1H, H-3 ^3^*J*_HH_ = 14.9, ^4^*J*_PH_ = 3.0 7.60, d 2H, m-Ph, ^3^*J*_HH_ = 8.7 7.19 d, 1H, H-4, ^3^*J*_HH_ = 14.9 6.92, d 2H, o-Ph, ^3^*J*_HH_ = 8.7 5.00 d, 1H, H-1, ^2^*J*_PH_ = 42.7 4.23 s, OMe	159.27 s, C-OMe 144.25 d, C-2, ^2^*J*_PC_ = 26.9 136.95 s, C-4 133.10 s, i-Ph 129.36 s, o-Ph 128.44 d, C-3, ^3^*J*_PC_ = 8.2 119.54 d, C-1, ^1^*J*_PC_ = 117.1 114.32 s, m-Ph 49.36 s, OMe	84.6 dd ^2^*J*_PH_ = 42.7 ^4^*J*_PH_ = 3.0
** 94 **	H	p-Cl-Ph	7.77 dd, 1H, H-3 ^3^*J*_HH_ = 14.5, ^4^*J*_PH_ = 2.7 7.63, d 2H, m-Ph, ^3^*J*_HH_ = 8.3 7.16 d, 1H, H-4. ^3^*J*_HH_ = 14.5 6.76, d 2H, o-Ph, ^3^*J*_HH_ = 8.3 5.63 d, 1H, H-1, ^2^*J*_PH_ = 41.2	143.09 d, C-2, ^2^*J*_PC_ = 27.6 139.00 s, i-Ph 136.29 s, C-4 134.60 s, C-Cl 129.24 s, m-Ph 129.18 s, o-Ph 128.51 d, C-3, ^3^*J*_PC_ = 7.8 119.34 d, C-1, ^1^*J*_PC_ = 116.9	88.7 dd ^2^*J*_PH_ = 41.2 ^4^*J*_PH_ = 2.7

**Table 11:** ^1^H, ^13^C and ^31^P NMR chemical shifts (δ, ppm) and coupling constants (*J*, Hz) of 1,5,2-diazaphosphorines **103**–**107**.

No.	Structure	^1^H	^13^C	^31^P
**103**	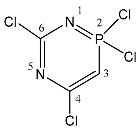	5.93 d, H-3 ^2^*J*_PH_ 14.0	163.15 d, C-4, *J*_PC_ 21.2 160.90 d, C-6, ^2^*J*_PC_ 6.2 88.02 d, C-3, *J*_PC_ 112.0	53.4 d ^2^*J*_PH_ 14.0
**104**	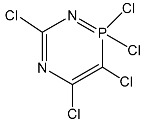	-	160.15 d, C-4, *J*_PC_ 37.8 157.73 d, C-6, ^2^*J*_PC_ 4.9 97.27 d, C-3, *J*_PC_ 132.8	44.7 s
**105**	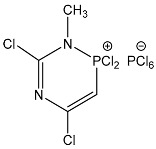	5.88 d, H-3 ^2^*J*_PH_ 42.2 3.45 d, CH_3_ ^3^*J*_PH_ 6.9	158.15 C-4, ^2^*J*_PC_ 23.4 156.90 C-6, ^2^*J*_PC_ 6.8 82.20 C-3, ^1^*J*_PC_ 116.0	64.4 dq ^2^*J*_PH_ 42.2 ^3^*J*_PH_ 6.9 –297.5 br s ${\mathrm{PCl}}_{6}^{-}$
**106**	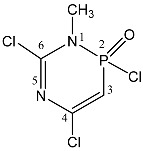	6.08 d, H-3 ^2^*J*_PH_ 12.8 3.38 d, CH_3_ ^3^*J*_PH_ 6.6	166.45 C-4, ^2^*J*_PC_ 24.2 159.70 C-6, ^2^*J*_PC_ 6.2 85.50 C-3, ^1^*J*_PC_ 112.0	21.5 dq ^2^*J*_PH_ 12.8 ^3^*J*_PH_ 6.6
**107**	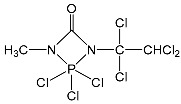	6.84 d, CHCl_2_ ^4^*J*_PH_ 1.0 3.10 d, CH_3_ ^3^*J*_PH_ 24.4	147.7 d, C=O, ^2^*J*_PC_ 17.5 95.8 d, C-Cl, ^2^*J*_PC_ 6.1 76.5 d, CHCl, ^3^*J*_PC_ 7.3 28.9 d, CH_3_, ^2^*J*_PC_ 2.5	−69.1 qd ^3^*J*_PH_ 24.4 ^4^*J*_PH_ 1.0

**Table 12:** ^1^H and ^31^P NMR chemical shifts (δ, ppm) and coupling constants (*J*, Hz) of 1,5,2-diazaphosphorines **108**–**114**.

No.	Structure	^1^H	^31^P
**108**	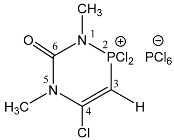	6.83 d, H-3, ^2^*J*_PH_ = 10.6 3.90 s, 5-CH_3_ 3.78 d, 1-CH_3_ ^3^*J*_PH_ = 9.9	58.3 dq ^2^*J*_PH_ = 10.6, ^3^*J*_PH_ = 9.9 −294.7 br s, ${\mathrm{PCl}}_{6}^{-}$
**109**	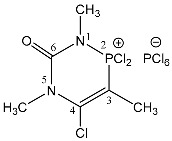	3.68 s, 5-CH_3_ 3.37 d, 1-CH_3_ ^3^*J*_PH_ = 9.6 1.85 d, 3-CH_3_ ^3^*J*_PH_ = 6.7	61.2 qq ^3^*J*_PH_ = 9.6, ^3^*J*_PH_ = 6.7 −297.6 br s, ${\mathrm{PCl}}_{6}^{-}$
**110**	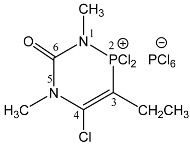	3.57 s, 5-CH_3_ 3.30 d, 1-CH_3_ ^3^*J*_PH_ = 12.2 2.85 dd, 3-CH_2_ ^3^*J*_PH_ = 10.0, ^3^*J*_HH_ = 8.2 1.75 d, 3-CH_3_ ^3^*J*_HH_ = 8.2	60.8 qt ^3^*J*_PH_ = 12.2, ^3^*J*_PH_ = 10.0 −297.9 br s, ${\mathrm{PCl}}_{6}^{-}$
**111**	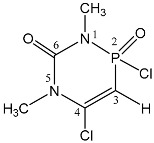	5.71 d, H-3 ^2^*J*_PH_ = 6.8 3.50 s, 5-CH_3_ 3.19 d, 1-CH_3_ ^3^*J*_PH_ = 7.3	17.1 qd ^3^*J*_PH_ = 7.3, ^2^*J*_PH_ = 6.8
**112**	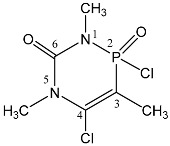	3.35 s, 5-CH_3_ 3.04 d, 1-CH_3_ ^3^*J*_PH_ = 7.6 1.96 d, 3-CH_3_ ^3^*J*_PH_ = 15.1	18.8 qq ^3^*J*_PH_ = 15.1, ^3^*J*_PH_ = 7.6
**113**	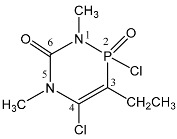	3.48 s, 5-CH_3_ 3.32 d, 1-CH_3_ ^3^*J*_PH_ = 10.0 2.85 dd, 3-CH_2_ ^3^*J*_PH_ = 9.0, ^3^*J*_HH_ = 7.8 1.75 d, 3-CH_3_ ^3^*J*_HH_ = 7.8	19.2 qt ^3^*J*_PH_ = 10.0, ^3^*J*_PH_ = 9.0
**114**	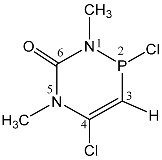	5.70 d, H-3 ^2^*J*_PH_ = 61.4 3.42 s, CH_3_ 3.14 d, CH_3_ ^3^*J*_PH_ = 14.2	100.5 dq ^2^*J*_PH_ = 61.4, ^3^*J*_PH_ = 14.2

**Table 13:** ^1^H, ^13^C and ^31^P NMR chemical shifts (δ, ppm) and coupling constants (*J*, Hz) of 1,5,2-diazaphosphorines **117** and **118**.

No.	Structure	^1^H	^31^P	^13^C
**117**	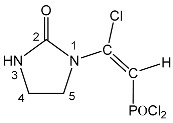	7.14 d, = CH ^2^*J*_PH_ = 21.1 6.38 br s, NH 4.10 t, 5-CH_2_ ^3^*J*_HH_ = 8.4 3.52 t, 4-CH_2_ ^3^*J*_HH_ = 8.4	27.9 d ^2^*J*_PH_ = 21.1	155.98 s, C = O 143.89 d, C-Cl, ^2^*J*_PC_ = 6.5 103.28 d, = CH ^1^*J*_PC_ = 176.4 45.75 s, 5-CH_2_ 36.38 s, 4-CH_2_
**118**	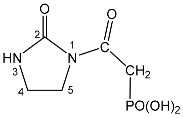	7.64 br s, NH 3.75 t, 5-CH_2_ ^3^*J*_HH_ = 8.2 3.57 d, CH_2_ ^2^*J*_PH_ = 21.8 3.28 t, 4-CH_2_ ^3^*J*_HH_ = 8.2	16.0 t ^2^*J*_PH_ = 21.8	165.70 d, C = O ^2^*J*_PC_ = 6.9 155.80 s, 2-C = O 42.21 s, 5-CH_2_ 35.33 s, 4-CH_2_ 35.28 d CH_2_ ^1^*J*_PC_ = 126.3

## Data Availability

Not applicable.
